# A lightweight deep learning framework for transformer fault diagnosis in smart grids using multiple scale CNN features

**DOI:** 10.1038/s41598-025-96290-2

**Published:** 2025-04-25

**Authors:** Omneya Attallah, Rania A. Ibrahim, Nahla E. Zakzouk

**Affiliations:** 1 Electronics and Communications Engineering Department, College of Engineering and Technology, Arab Academy for Science, Technology, and Maritime Transport, Alexandria, 21937 Egypt; 2 Wearables, Biosensing, and Biosignal Processing Laboratory, Arab Academy for Science, Technology, and Maritime Transport, Alexandria, 21937 Egypt; 3 Electrical and Control Engineering Department, College of Engineering and Technology, Arab Academy for Science, Technology, and Maritime Transport, Alexandria, 21937 Egypt

**Keywords:** Trans-light approach, Smart fault diagnosis, Power transformer, Inter-turn faults, Infra-red (IR) thermal imaging, Deep learning (DL), Convolutional neural network (CNN), Feature extraction and selection, Electrical and electronic engineering, Power distribution

## Abstract

Scheduled maintenance and condition monitoring of power transformers in smart grids is mandatory to reduce their downtimes and maintain economic benefits. However, to minimize energy losses during inspection, non-invasive fault diagnosis techniques such as thermogram imaging can enable continuous monitoring of transformer health with minimal out-of-service time. Deep learning (DL) has proven to be a fast and efficient intelligent diagnostic tool. In this paper, a DL-based thermography method is proposed called Trans-Light for transformers’ interturn faults detection and short-circuit severity identification. Trans-light extracts deep features from two deep layers of a convolutional neural network (CNN) rather than depending on one layer, thus obtaining more intricate patterns. Moreover, a Dual-tree Complex Wavelet Transform method is adopted which offers two enhancements. First, it acquires time–frequency knowledge besides the already obtained spatial information and second, it reduces the huge deep features dimensionality. Trans-light combines extracted deep features, then a feature selection process is applied to further reduce features’ size, thus decreasing computation burden and reducing classification and training time. To validate the proposed scheme’s diagnosis performance and robustness, different combinations of two CNN models, two feature selection methods, and six classifiers were tested, applying the proposed Trans-light framework, under noise-free and noise-existing conditions. Experimental results indicated that the combination of the LDA classifier, applied with the ResNet-18 CNN model and trained with merged deep features undergoing the chi-square (χ^2^) selection approach, attained superior performance under noise-free conditions. Compared to its counterparts in previous work, this configuration outperforms their performance since it uses the fewest features’ number yet maintains 100% classification accuracy. Besides, it attained robust performance under two different noise natures again with minimal features’ dimension, thus minimizing computational load and implementation complexity.

## Introduction

Power transformers are regarded as a pivotal asset within smart grids facilitating electricity distribution to consumers. Any failure in this crucial element leads to a power outage which could trigger substantial consequences for utilities and electrical shortages to end-users^1^. Various load sectors are affected by power interruptions and transformers’ failures which are translated into Customer Interruption Costs (CIC) that depend on the class of the load sector, and interruption scale and duration^2^. A major failure in transformers is defined as any situation that requires the transformer to be removed from service for a period longer than seven days for investigation, remedial work, or replacement to restore it to the initial service capability^3^. This greatly disrupts the operations of businesses and industries which have profound economic implications.

Therefore, continuous condition monitoring of the transformer’s health as well as early detection of its expected upgoing faults is essential to ensure the transformer remains in optimal and healthy condition. Moreover, the identification of fault class, scale, and duration assists in enhancing maintenance schedules, manufacturing power transformers, and estimating the CICs as well as maintaining the energy system’s reliability and security^1,2^. Fault classifications in power transformers can occur in different components, including windings, tap changers, core, tank, bushing, or auxiliaries. As per CIGRE and IEEE statistical surveys, reported in^4,5^, Table 1 provides percentages of occurrences for different types of transformer failures, revealing that winding failures are regarded as the most common to occur among all surveys.

**Table 1:** CIGRE and IEEE surveys for transformer components faults percentage^4^.

Fault type	Winding fault	Tap changer	Bushing	Auxiliary	Core	Tank
CIGRE, %	37.69	31.16	17.16	10.63	2.61	0.75
IEEE survey %	41	–	13	17	10	3

In distribution side transformers, primary and secondary side windings withstand excessive dielectric, thermal, mechanical, and electrical stress after a period of prolonged operation^6^ or during the manufacturing process^7^. This may result in winding turn-to-turn short circuits when two or more adjacent coils come into direct contact due to insulation layer damage because of these stresses. As a result, high circulating current flows through the faulted windings, creating localized hot spots near the faulted area, which further degrades the winding insulation due to electrothermal effects, leading to total failure^8,9^. Although the short circuit currents due to winding faults at an emerging level may not lead to immediate failure, they can reduce the winding’s ability to endure mechanical/electrical stresses. Hence, early detection of an inter-turn transformer fault decreases the probability of fatal failures that may happen in the near future^10^.

Much research has been conducted on methods for condition monitoring and detecting short circuit faults in transformer windings, which can be widely categorized as offline and online techniques^11,12^. Off-line techniques refer to methods that can only be conducted during maintenance while the equipment is out of service. Whereas online monitoring and diagnostic tools enable fault detection without power supply disruptions during the detection process^13^. Hence, online techniques outweigh offline ones as a non-destructive technique capable of continuous condition monitoring without interrupting transformer operation as well as fault detection at the earliest stage before system failure Thus, power supply is maintained, service interruption is minimized, and energy saving is maximized.

Thermography Analysis (TA) is a standard online non-invasive diagnostic tool with significant potential in identifying early equipment faults^14^. As an electrical device degrades, both its thermal energy and resistance increase, and IR radiation is emitted according to the device’s temperature and emissivity. TA detection method is based on capturing heat patterns and analyzing thermal IR images of electrical components since the heat rise can help identify the damaged part of the component by its level of seriousness^14–19^ Although relatively expensive, being based on high-resolution IR cameras^20^, they minimize the concerns and problems associated with sensors’ proximity and location due to their non-contact integrated feature.

After detecting heat patterns in the captured IR images, fault-related features must be extracted for fault diagnosis and prognosis. With the rapid advancement in artificial intelligence (AI), Machine Learning (ML)-based techniques are quite appealing for automatic accurate fault symptom extraction in electrical equipment^21^. These methods feature robust and efficient response, high adaptational capabilities, and do not require full prior data which may be practically hard to obtain^22^. However, when handling a huge amount of data, traditional ML methods may encounter some limitations which can be overcome using the recently developed deep learning (DL) approaches^23–25^. Rather than artificial feature extraction in traditional ML techniques, DL approaches can automatically learn fault features from the collected data. Hence, they attempt to provide end-to-end diagnosis models with high accuracy when handling the increasingly grown data^24^ Among DL approaches, convolutional neural network (CNN)-based approaches are commonly used for image analysis serving as an efficient tool for incipient fault identification with very high accuracy^26^.

Although image processing techniques, especially deep learning-based ones, offer potential advantages for transformer condition monitoring, including non-invasive, accurate, and fast inspection, they face general limitations that affect diagnosis accuracy and complexity as follows:

- Challenges in data acquisition: These include data imbalance, especially for insulation faults as they are rare to happen^27^, and overfitting when the available data is not sufficiently large or diverse^28^. This can reduce the model’s generalization capability, making it unreliable when exposed to new operating conditions or fault scenarios.
- *Computational resources and cost* Image processing techniques, especially DL-based methods with multiple-layer models and large datasets, mandate substantial computational load, time-consuming training, and resource-intensive needs requiring high-end GPUs or cloud computing infrastructure^29^. This makes real-time or on-site monitoring difficult, and costly, especially in resource-limited environments^30^
- *Concept drift, model updates, and maintenance* In dynamic environments, transformer conditions may change over time due to aging, wear, or external environmental factors and noises; thus regular retraining with new data to maintain performance is necessary^31^. However, this can be costly, labor-intensive, and time-consuming, particularly in models that include big feature dimensions for training and classification.

Besides these general limitations, after analyzing ML-based and DL-based fault diagnosis approaches, developed in the literature, for transformer interturn fault detection based on IR images^14,17–19^, several gaps were identified. First, those which employed ML techniques,^14,17^, relied on manual feature extraction methods which are complex processes and prone to error. On the other hand, those employed DL,^18,19^, performed either end-to-end DL classification or extracted deep features of huge dimensions. Nevertheless, end-to-end classification is very time-consuming and complex while huge deep features’ extraction increases the complexity of detection and diagnosis, thus feature selection is essential. Moreover, these deep features just represent the spatial representation included in an image, however, acquiring a time–frequency demonstration besides a spatial one usually enhances the detection procedure^32^. Additionally, these studies retrieved features from one deep layer only, nonetheless, studies carried out by researchers^33,34^ have demonstrated that various layers of a CNN possess the capability to gather distinct categories of knowledge. Finally, some of the studies in the literature relied on segmentation/clustering steps to achieve the detection and diagnosis procedure, yet these steps increase system complexity.

Hence, to mitigate the pre-discussed general limitations and to close the aforementioned research gap, this paper proposes an online non-invasive five-stage DL-based thermography method for transformer winding fault detection and classification. This method serves as a smart CNN-based fault detection tool that maximizes energy savings due to its high accuracy, low computational burden, fast detection of incipient faults, and lack of interruption to transformer operation. Enhancements and contributions of the suggested framework are as follows:

- The absence of any segmentation or clustering phases which adds to system compactness
- In the first stage, augmentation methods are carried out to increase the number of training images, thus preventing the network from falling into the overfitting issue and improving DL training performance.
- In the second stage, lightweight CNNs are used based on transfer learning which is useful in CNNs used in computer vision tasks. It uses models that have already been trained, thus speeding up and improving training procedures.
- In the third stage, Dual-tree Complex Wavelet Transform (DTCWT) is utilized to acquire time–frequency data besides spatial knowledge instead of relying solely on spatial data, thus improving diagnosis accuracy and data extraction. Moreover, DTCWT has the merit of lowering the dimensionality of large-size deep features which in turn simplifies computation complexity.
- Besides extracting deep features from the CNN pooling layer through DTCWT, others are extracted from CNN fully connected layers. Deep features acquired from both layers are merged to benefit from each layer with its extinct knowledge, thus enhancing diagnosis performance and accuracy.
- In the fourth stage, merged deep features pass via the feature selection phase, removing unnecessary or duplicate attributes, to further diminish features’ dimensions, thus reducing model complexity and training time.
- In the fifth stage, six ML classifiers, along with two feature selection approaches are tested with two different CNN models to determine the combination that achieves the highest diagnosis performance with the least feature size under noise-free conditions. After analyzing the experimental result, it was concluded that the LDA classifier deployed with the ResNet-18 CNN model and trained with merged deep features undergoing the chi-square (χ^2^) selection approach, attained the best performance of 100% accuracy with only 50 features. Thus, the proposed framework outweighs other research studies, which applied the same dataset and attained high accuracies, yet at the cost of quite more feature count and more complex implementation.
- Moreover, experimental tests are repeated under two different noise types to evaluate the proposed system’s robustness. Again, the proposed combination (LDA classifier with ResNet-18 CNN model and χ^2^ approach) achieved second place among other combinations in terms of diagnosis accuracy with the least number of features.

In short, the proposed configuration achieves the best compromise, under noise-free and noise-existing conditions regarding diagnosis performance with the least feature count, compared to its counterparts, resulting in elevated classification accuracy with reduced computational complexity.

## Related work

The most recent related work involving transformers’ fault detection based on IR thermography technology is presented in Table 2. Studies in^35–37^ used different image-processing techniques such as Robert’s algorithm, Otsu thresholding, Canny edge detection, and image segmentation for fault detection in thermal images. Although these techniques provide quantitative metrics related to fault detection and are robust to variations in thermal image quality, they often require manual feature extraction and high complexity, resulting in high processing time. Consequently, their performance may be limited with complex imagery and dynamic datasets. The study in^38^ demonstrates the applicability of using acoustic emission and IR thermography for oil transformer testing for condition monitoring and early fault detection. IR thermography helped predict the point of highest temperature inside the transformer core and windings, while acoustic emission was able to detect partial discharge contributing to improved maintenance practices and operational reliability. Both methodologies reveal results that can be used to complement one another. The work in^16,39,40^ applied artificial neural networks (ANN), Adaptive Neuro-Fuzzy Inference System (ANFIS), and fuzzy fault diagnosis models respectively for fault diagnosis of substation and distribution transformer health status. In^1^, an ANN and Fuzzy Inference System were designed to interpret thermal infrared images and correlate them with insulating oil analysis results. The study in^2^ focused on the analysis of IR images at a remote terminal unit by extracting Speeded-Up Robust Features (SURF) which are further processed by an ANFIS system in conjunction with an SVM classifier in a real-time manner. A fuzzy fault diagnostic model was designed in^40^ to handle uncertainties of data obtained from multiple sensors for a more efficient fault location detection. The study of^41^ employed image processing, graph-based semi-supervised learning, and Generative Adversarial Network (GAN) for the detection of overheating faults and equipment defects with a recorded accuracy ranging between 82.2% and 86.2%. The evolution of DL models for anomaly detection in transformers was applied in various studies such as^42–44^ where classification accuracies ranged from 96% in^36^ to 99.95% in^44^

**Table 2:** Summary of related Thermography and IR-based techniques developed for transformer fault detection.

#	Fault type and classes	Measured signals	Fault diagnosis method	Results
^16^	Incipient overheating faults in transformers # of classes: 2	IR temperature and dissolved gas analysis	Artificial neural networks and Adaptive Neuro-Fuzzy Inference System	ANN accuracy: 86% ANFIS accuracy: 83%
^39^	Substation equipment # of classes: 14 test cases	IR images	Adaptive neuro-fuzzy Inference System (ANFIS) and Support Vector Machine (SVM) classifier	Results for SVM were superior to ANFIS Undefined accuracy
^42^	Thermal monitoring of cabinet temperature for distribution transformer # of classes: undefined	IR images	Deep Learning with CNN	Undefined accuracy
^35^	Three-phase distribution transformers # of classes: undefined	IR images	Image processing is based on Otsu thresholding, Canny edge detection, image segmentation, noise reduction, histogram equalization, and feature extraction	Undefined accuracy
^40^	Power transformer hot spots # of classes: 10	Multi-IR images and discharge signals using a discharge circuit detection module	Fuzzy fault diagnosis model with the exponential trust function	The fuzzy exponential trust function reduces the standard deviation of the fusion result by 20.52% compared to the Arithmetic Mean Approach and by 10.62% compared to the General Trust Function method
^38^	Distribution transformer hot spots and partial discharge # of classes: undefined	Acoustic emission and IR images	For thermal analysis, temperature ranges were obtained through a thermal camera for the transformer surface	Undefined accuracy
^14^	Transformer inter-turn faults # of classes: 9	IR images	ML for feature extraction + Decision Tree and Random Forest (RF) as classifiers	Accuracy: 95.6%
^43^	Defective and non-defective HV transformer # of classes: 2	IR images	Deep learning using pre-trained AlexNet. Random RF and SVM were trained for classification	Accuracy RF: 96% Accuracy SVM: 90%
^36^	Top-oil and radiator temperatures # of classes: undefined	Thermal model, thermography method, and computational fluid dynamic	Image processing techniques to determine radiator hot spots and top-oil temperature	Undefined accuracy
^44^	Cast-resin transformer # of classes: 9	IR images	Wasserstein Autoencoder Reconstruction (WAR) model and the Differential Image Classification (DIC)	WAR-DIC results (for all classes): Accuracy: 99.95%
^37^	Substation transformer # of classes: undefined	IR images	Weighted Average algorithm to process gray-scale image using improved Robert’s algorithm	Undefined accuracy
^41^	Overheating faults and equipment defect detection # of classes: undefined	IR images	Image processing, graph-based semi-supervised learning, and Generative Adversarial Network (GAN)	Overheating accuracy: 82.2% Equipment defect accuracy: 86.2%
^17^	Transformer inter-turn faults # of classes: 9	IR images	GIST feature extraction then ML with different classifiers where SVM shows the best classification	Accuracy with SVM: 100% Time: 8.924 s
^18^	Transformer inter-turn faults # of classes: 9	IR images	Different deep learning architectures (Dense-Net201, MobileNetV2, ResNet50, ShuffleNet, Xception) are examined	All architectures can achieve 100% accuracy AVR-based evaluations: ShuffleNet is the most robust Computation time-based evaluations: ShuffleNet fastest
^19^	Transformer inter-turn faults # of classes: 9	IR images	VGG16, Inception V3, ResNet models compared to Crafted CNN tailored to the studied dataset	VGG16: Accuracy 90.9% Inception V3: 86.36%, CNN accuracy: 100%

In ^14,17–19^, the work main focused on the detection of transformer interturn faults and identifying different short circuit severity. In ^14^, authors have established a dataset considering one healthy and seven different cases of short circuit failures in the common core winding of a single-phase transformer. To distinguish the latter, a pre-processing stage was carried out which divided the data into two categories based on their thermal condition, followed by the utilization of Interpretable Machine Learning techniques. However, 95.6% classification accuracy was attained. Utilizing the same dataset introduced in^14^, authors of^17^ employed GIST feature extraction coupled with an SVM classifier for fault classification achieving 100% accuracy. Similarly, the work in^18^ attained 100% accuracy, with the same dataset, by applying different pre-trained deep learning architectures. Accordingly, mini-batch size, learning rate, learning rate drop factor, and optimizer type are evaluated to perform the transfer learning task to reveal the most appropriate model for thermal image classification. ShuffleNet is marked as the fastest, most robust, and the highest in terms of average success rate from all trials. Finally,^19^ based their work on the same dataset found in^14^ where two methodologies were compared. The first involved a hand-crafted CNN while the second involved a transfer learning approach. Results showed accuracy ranging from 90.9% and 86.36% with pre-trained models, while the CNN model achieved 100% accuracy. Despite achieving 100% accuracy in^17–19^, authors relied on a huge dimension of extracted features reaching up to 2048 features which in turn increases classification complexity and training time. Thus, modifications proposed in this paper contribute to reducing features’ size to simplify model implementation and reduce computations burden while maintaining 100% accuracy.

## Transformers dataset description

**Fig. 1:**
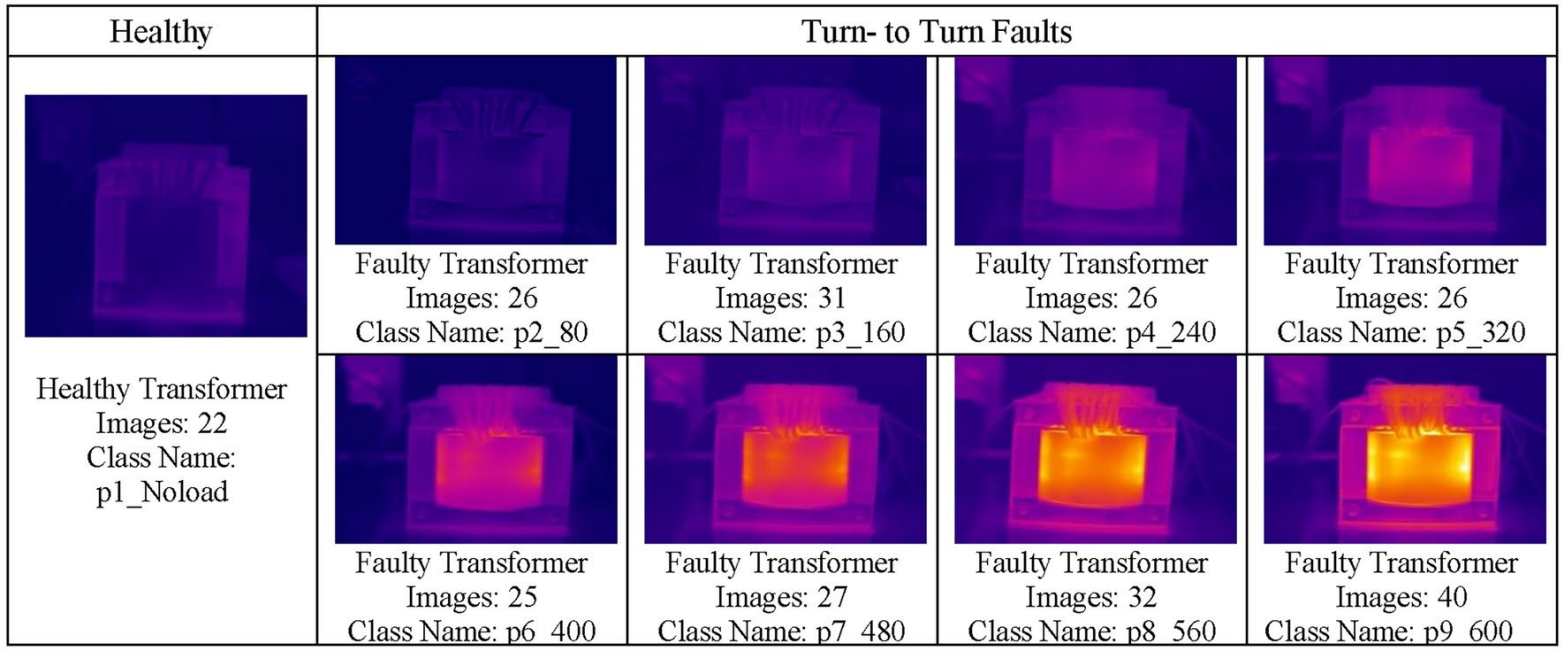
Dataset under consideration: condition, number of images per class and class labels.

The employed dataset in this study was developed in^14^. Thermal imaging acquisition was performed using a Dali-tech T4/T8 infrared thermal image camera at an ambient temperature of 23°. All experiments were conducted on a 1kW, 220 V, 50 Hz, single-phase transformer. The comprised dataset involves nine classes all associated with transformer operation, with one healthy state and 8 turn- to- turn faults at different short circuit levels in common core winding with a total of 255 captured images as shown in Fig. 1. Faulty classes, categorized by type, severity, and short-circuit (SC) rounds are listed according to Table 3. Severity reflects the short circuit percentage of the windings which yields a heat change in thermal image concerning the short-circuit ratio.

**Table 3:** Image counts per class, fault severity, and SC rounds for different transformer faults at NO LOAD.

Class name	Type	Severity (%)	No. of SC rounds	Number of images
p1_Noload	–	–	–	22
p2_80	Turn- to—Turn	13	80	26
p3_160	Turn- to—Turn	26	160	31
p4_240	Turn- to—Turn	40	240	26
p5_320	Turn- to—Turn	53	320	26
p6_400	Turn- to—Turn	66	400	25
p7_480	Turn- to—Turn	80	480	27
p8_560	Turn- to—Turn	93	560	32
p9_600	Turn- to—Turn	100	600	40

## Proposed five-stage DL-based framework

This study proposes a novel noncontact and noninvasive DL-based framework for transformer fault detection and diagnosis to analyze an IR images-based dataset. The proposed framework has five stages as follows:

i. IR image preparation
ii. Lightweight DL model implementation
iii. Two-fold layer feature extraction and time–frequency representation
iv. Deep feature incorporation and selection
v. Fault detection and diagnosis.

**Fig. 2:**
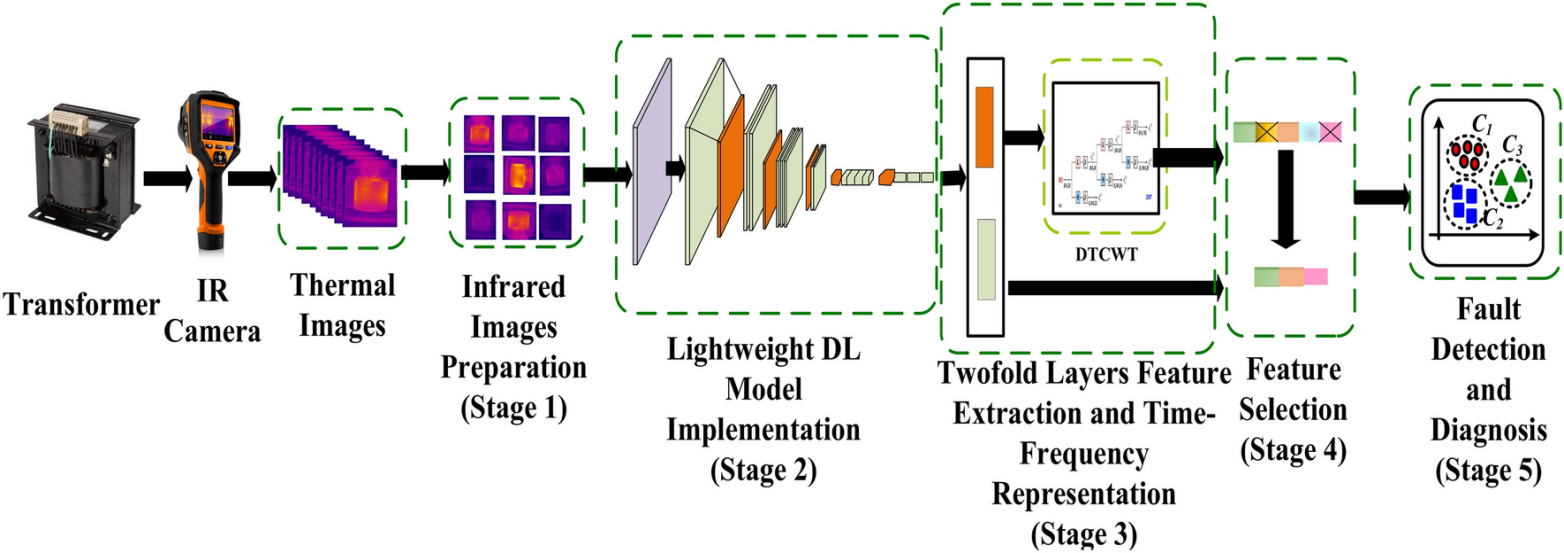
Structure of the proposed five-stage DL-based framework for transformer fault detection.

Initially, the IR image dimensions are modified, and multiple augmentation techniques are subsequently adopted to increase the number of images. Next, two lightweight DL models are implemented using transfer learning. Afterward, deep features are retrieved from two different deep layers of each CNN involving pooling and fully connected layers, followed by DTCWT applied to pooling features to show the time–frequency representation of the deep features. DTCWT is also applied to reduce the size of the deep feature vectors. After that, the deep features of both layers for each DL model are combined, and a feature selection process is applied to choose among the most influential deep features. Finally, several machine learning classifiers are employed to select the most effective one for transformer faults’ detection and diagnosis. The suggested framework with the pre-discussed stages is demonstrated in Fig. 2.

### Infrared image preparation

IR image aspects of the considered dataset are first changed to match the dimensions of the input layer of each DL model, which is equal to 224 × 224 × 3. Next, the dataset is split into 80–20% training and validation sets. After that, the training images are augmented via multiple augmentation procedures. Augmentation is a group of techniques used to increase the number of training images to prevent the network from falling into the overfitting issue and improve the performance of DL training. The augmentation methods utilized in this study include flipping, rotation, shearing, transformation, and scaling^45^. Details of these augmentation methods are displayed in Table 4.

**Table 4:** Augmentation procedure details.

Augmentation procedure	Range
Flipping both vertically and horizontally	− 50 to 50
Rotate both horizontally and vertically	− 70 to 70
Scaling	0.5 to 2.5
Shearing	− 60 to 60

### Lightweight DL model implementation

In this stage, two lightweight CNNs, MobileNet and ResNet-18, are implemented via transfer learning. Transfer learning (TL) is a useful form of DL that uses models that have already been trained to speed up and improve the training procedure^1,46^. This is especially true for CNNs that are used in computer vision tasks. Through the use of a pre-trained CNN that was first trained on a sizable dataset such as ImageNet for general image classification, TL enabled researchers to take advantage of the model’s built-in feature extraction capacities. After learning these attributes from an enormous quantity of data, they can be used on a new task with a lesser quantity of data that is specific to that task. This method has many benefits, such as reducing the training duration and processing power, and might even help the model being targeted perform better on tasks with minimal data^47^. This approach is quite helpful when attaining large datasets with labels might be costly or take considerable time. In this study, TL is used to adapt the number of fully connected layers of each CNN to 9, which is equivalent to the number of fault classes in the dataset. Then, several CNNs’ hyperparameters are modified, as described in the Experimental Implementation section. Afterward, the infrared images are fed to the lightweight CNN to start the retraining procedure.

### Two-fold layer feature extraction and time–frequency representation

Studies carried out by researchers^33,34^ have demonstrated that various layers of a CNN possess the capability to extract distinct categories of knowledge. A CNN begins to acquire more intricate patterns as it passes through its layers. During the initial phases, the DL model identifies fundamental components such as edges and textures, while in the later stages, it gains an understanding of fault-specific and complex patterns. Hence, extracting deep features from more than one layer in each CNN is beneficial. Thus, once the retraining process of the lightweight CNNs is complete, TL is reutilized again in this stage to obtain features from the last pooling and fully connected deep layers.

Although pooling layers are considered to reduce the dimensionality of the feature maps, the features obtained from these layers may still be large. Moreover, features acquired from CNNs reveal spatial information from the input data only; however, attaining time–frequency information usually enhances diagnostic performance. Therefore, the dual-tree complex wavelet transforms (DTCWT) technique is employed in this study because it is a multipurpose method that can reduce dimensionality and convey time–frequency demonstrations. DTCWT has become an important method because it can perform well in both the frequency and time domains and has built-in benefits for feature reduction. The DTCWT is different from the conventional discrete wavelet transform (DWT) because it uses a pair of parallel filter banks, which ensures that the near shift is not affected and that it can pick out image features more accurately^48^. Moreover, the complex nature of the DTCWT coefficients allows phase information to be included, which provides a more complete picture of the signal than real-valued DWT^49^.

Conclusively, using the DTCWT, beneficial data can be obtained from IR images by combining time–frequency analysis with spatial information, enhancing diagnostic performance. According to its feature reduction capability, DTCWT is quite promising. Unnecessary or noisy data can be omitted by examining the sub-bands created by this transform, which decreases the feature set. This approach not only accelerates computations but also helps models work effectively in tasks such as classification or segmentation^48^.

It is important to note that the two stages of breakdown from the DTCWT were used. The low pass coefficients from the second level were selected as the decreased attributes. Table 5 shows the dimensions of the features acquired from every layer of the two CNNs. It is concluded that feature dimensions obtained from the pooling layer after applying the DTCWT are less than those before applying this transform. Thus, besides being employed to uncover the spatial-time–frequency representations of pooling layer features, DTCWT is used to condense these features’ dimensions.

**Table 5:** The dimensionality of the feature space obtained from the two layers of each CNN.

Model	Pooling layer	Pooling layer (after DTCWT)	Fully connected Layer
ResNet-18	512	256	9
MobileNet	1280	640	9

### Deep feature incorporation and selection

The two levels of deep features obtained from both layers (pooling layer after DTCWT + fully connected layer) are then incorporated in a concatenated manner. Feature selection is then adopted to select among these deep features the most influential features and remove redundant and irrelevant features.

Generally, feature selection is crucial in finding meaningful patterns from data that have a large number of dimensions and removing unnecessary or duplicate attributes, which leads to a reduction in the risk of overfitting and an improvement in model generalizability^50,51^. Integrating a feature selection procedure, in transformer fault diagnosis technique, can significantly enhance model interpretability owing to its capabilities and inherited merits^52,53^. Feature selection is crucial for enhancing the interpretability of the suggested framework. Decreasing the size of the deep features simplifies the model, making it less complex and more amenable to analysis.

In this study, chi-square (χ^2^) and ReliefF feature selection approaches were used to search among the combined double layers of deep features for the most influential features. The χ^2^ feature selection method is commonly used for selecting significant attributes within datasets with a large number of dimensions by utilizing the χ^2^ statistic to evaluate the lack of dependency across an attribute and the class label. Variables that have a strong connection or dependence on the class label are assigned greater χ^2^ scores, demonstrating their potential importance for classification or prediction problems^54^. In contrast, features that have low χ^2^ values indicate a minimal connection with the label and are regarded as less useful. Researchers can enhance the generalizability of a model by choosing variables with high χ^2^ scores, which leads to a more concise feature set and reduces model complexity. On the other hand, ReliefF^55^ is a methodology for feature selection that determines pertinent attributes by assessing their significance in distinguishing between instances of identical and disparate classes. This method allocates weights to variables by progressively assessing their capacity to differentiate adjacent observations. For every attribute, the method assesses its significance by contrasting the feature values of one particular case with those of its closest neighbors within the same class (nearest hit) and across various categories (nearest miss). It can become computationally demanding when utilized with extensive datasets characterized by multidimensional feature space.

The χ^2^ feature selection technique was selected for its straightforwardness and efficacy in diminishing the dimensions of the deep features derived through the CNN layers. This approach assesses the statistical dependency between every attribute and the class of interest, rendering it especially appropriate for classification tasks involving categorical data, including transformer fault detection and diagnosis. The principal justifications for choosing χ^2^ versus other approaches are outlined below:

1. The χ^2^ test is less computationally demanding than more intricate methodologies such as mutual information or recursive feature elimination (RFE). Due to the extensive deep features derived from the CNN layers, computational effectiveness was essential to guarantee that the classification model would be trained and assessed within acceptable time limits.

2. The χ^2^ statistic yields transparent and comprehensible results by prioritising features according to their significance to the target class. This interpretability is essential for identifying the features that most significantly influence fault detection and diagnosis, thereby improving the model’s transparency.

3. In contrast to RFE, which necessitates repetitive model training with various feature subsets, χ^2^ can evaluate all attributes concurrently, rendering it suitable for datasets with large dimensions.

Although mutual information and RFE are potent techniques, they were excluded due to their elevated computational requirements and complexity, which possibly impeded the overall framework without offering substantial additional advantages in this particular application.

### Fault detection and diagnosis

The last stage of the framework involves fault detection and diagnosis. For this stage, six machine learning models were utilized: linear discriminate analysis (LDA), k-nearest neighbor (kNN), linear support vector machine (L-SVM), cubic support vector machine (C-SVM), quadratic support vector machine (Q-SVM), and medium Gaussian support vector machine (M-SVM). The efficacy of classifiers was determined through k-fold validation (k = 10), where the data were randomly separated into k subsets for training and testing purposes. The classification algorithm is constructed by dividing the learning procedure into k − 1 sections, with the remaining k proportions used for testing to estimate the accuracy of the model. This process is iterated, with each iteration using k − 1 folds for training and the remainder of the k folds for testing.

## Experimental settings

In this section, the settings of the experimental work are demonstrated.

### Parameters fine-tuning

Hyperparameters and specifications of the proposed framework include the following;

- The minibatch size of 5, which determines the volume of information to be utilized to perform weight updates within every sub-epoch, was determined following an assessment of various batch sizes. Experiments carried out in this study, demonstrate that smaller minibatches produced superior generalization performance which is consistent with existing literature on limited data scenarios.
- The learning rate was established at 0.00001. This value was established through repeated testing, optimizing rapid convergence while preventing overshooting the optimal point. Elevated learning rates resulted in instability, whereas diminished rates induced excessively gradual convergence.
- The total amount of training epochs was established at 50, as additional epochs demonstrated no substantial enhancement in performance. This decision was substantiated by observing the loss function and confirming the absence of overfitting during training.
- Regarding the optimization method, the models were developed utilizing stochastic gradient descent (SGD) in conjunction with the momentum technique. This optimization method was selected to accelerate convergence and avoid entrapment in local minima, thereby increasing the resilience of the learning step.
- The number of k values for the kNN was 1, and the Euclidean distance was used to determine the neighbors.
- Every experiment was carried out on a system that had an Intel(R) Core (TM) i7-10750H processor running at 2.6 MHz. A 64-bit operating system and an NVIDIA GeForce GTX 1660 graphics card with 6 GB of RAM were additionally contained in the framework. MATLAB 2022b was used to carry out experiments, offering the required tools for data analysis, designing models, and performance assessment, through toolboxes employed to construct the framework; image processing, deep learning, and statistics.
- Different machine learning classifiers were tested for their capability in fault detection and classification:
- LDA classifier was selected for its distinct benefits relevant to the considered application. Such classifiers are adept at managing reduced-dimensionality feature spaces, which corresponds to the targeted framework’s feature-size reduction objective employing DTCWT and feature selection methods.
- kNN classifier provides simplicity and resilience, especially for datasets exhibiting clear patterns, and its efficacy is enhanced by the optimized feature set.
- SVM classifier was used as well, for its capacity to manage both linear and nonlinear decision boundaries through kernel functions, offering adaptability in identifying intricate fault patterns. Its regularization ability mitigates overfitting, which is especially crucial when utilizing limited, high-quality features. The efficacy of SVM in using the discriminative strength of the diminished feature set guarantees elevated accuracy and resilience, even in difficult classification challenges.

### Evaluation measures

The initial factor to consider, when addressing classification problems, is frequently ‘accuracy’, which is a basic statistic that reflects the percentage of correctly categorized cases. Nevertheless, accuracy is susceptible to deactivation in imbalanced datasets, as the model may perform well in identifying the majority class but overlooks the minority class. During these circumstances, it is of utmost importance to prioritize ‘Precision’, which reflects the positive predictive value; ‘Sensitivity’, which reflects the true positive rate; and ‘Specificity’, which reflects the true negative rate. Furthermore, the F1-score combines precision and sensitivity (recall) and offers an equitable assessment of the model’s performance. These metrics are determined using Eqs. (1–5) as follows:

1 $$Accuracy = \left( {TP + TN} \right)/\left( {TN + FP + FN + TP} \right)$$

2 $$Sensitivity = TP/\left( {TP + FN} \right)$$

3 $$Specificity = TN/\left( {TN + FP} \right)$$

4 $$Precision = TP/\left( {TP + FP} \right)$$

5 $$F1 - Score = \left( {2 \times TP} \right)/\left( {\left( {2 \times TP} \right) + FP + FN} \right)$$

## Experiment implementation and results

The proposed diagnosis stages are implemented in three contexts to confirm each stage’s effectiveness:

- In Context I, deep features acquired from the fully connected layer of each CNN in addition to the DTCWT features obtained from the pooling layer are used to independently feed the six ML classifiers to validate the effectiveness of DTCWT.
- In Context II, the deep features of both deep layers are combined and then used to train the considered classifiers to verify the effect of integrating features from more than one CNN layer on classification performance.
- In Context III, combined dual deep layers’ features are selected using the considered feature selection approaches. Then, the selected features are fed to the six classifiers to evaluate the importance of feature selection.

### Context I results

This section displays the results of the six ML classifiers trained separately using DTCWT features obtained from the pooling layer of each CNN (spatial-time–frequency features) and fully connected features. Results shown in Table 6 clarify that, for MobileNet CNN, features attained using DTCWT and fully connected features have comparable performances except for the LDA classifier, which achieved better performance using the fully connected features. This is obvious as the accuracy reached 98.8% and 99.6% for LDA, 99.2% and 99.2% for kNN, 97.6% and 97.6% for LSVM, 98.8% and 98.8% for QSVM, 98.4% and 98.4% for CSVM, and 98.8% and 98.8% for MSVM using DTCWT and fully connected features, respectively. Nevertheless, for ResNet-18, the classifiers trained with DTCWT features achieved higher accuracy than those trained with fully connected features, except for MSVM as shown in Table 6. Accuracies attained with DTCWT features were 99.6%, 97.6%, 97.6%, 98.4%, and 99.6% versus 97.6%, 96.5%, 96.5%, 97.3% and 98.0% attained by fully connected features for LDA, kNN, LSVM, QSVM, and CSVM respectively. Oppositely, MSVM achieved 95.3% accuracy with DTCWT, which was less than the 96.9% accuracy achieved with the fully connected features.

**Table 6:** Diagnosis accuracy (%) using six classifiers trained with DTCWT features and fully connected features.

	Accuracy (%)					
Features	LDA	kNN	LSVM	QSVM	CSVM	MSVM
MobileNet						
DTCWT	98.8	99.2	97.6	98.8	98.4	98.8
Fully-connected	99.6	99.2	97.6	98.8	98.4	98.8
ResNet-18						
DTCWT	99.6	97.6	97.6	98.4	99.6	95.3
Fully-connected	97.6	96.5	96.5	97.3	98.0	96.9

Conclusively, in most cases, training different classifiers with DTCWT features gave high diagnosis accuracies. Thus, DTCWT’s effectiveness is verified since it acquires time–frequency information as well as spatial knowledge instead of relying on spatial data alone besides its merit of lowering the dimensionality of large-size deep features.

### Context II results

**Fig. 3:**
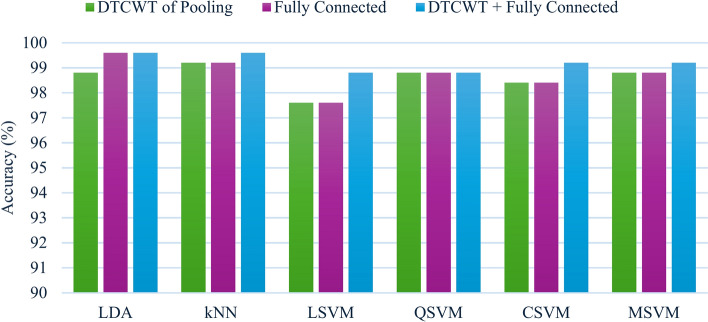
Accuracy results of the MobileNet CNN for the six classifiers considered when trained using features obtained from the pooling layer, fully connected layer, and combined DTCWT and fully connected layer.

**Fig. 4:**
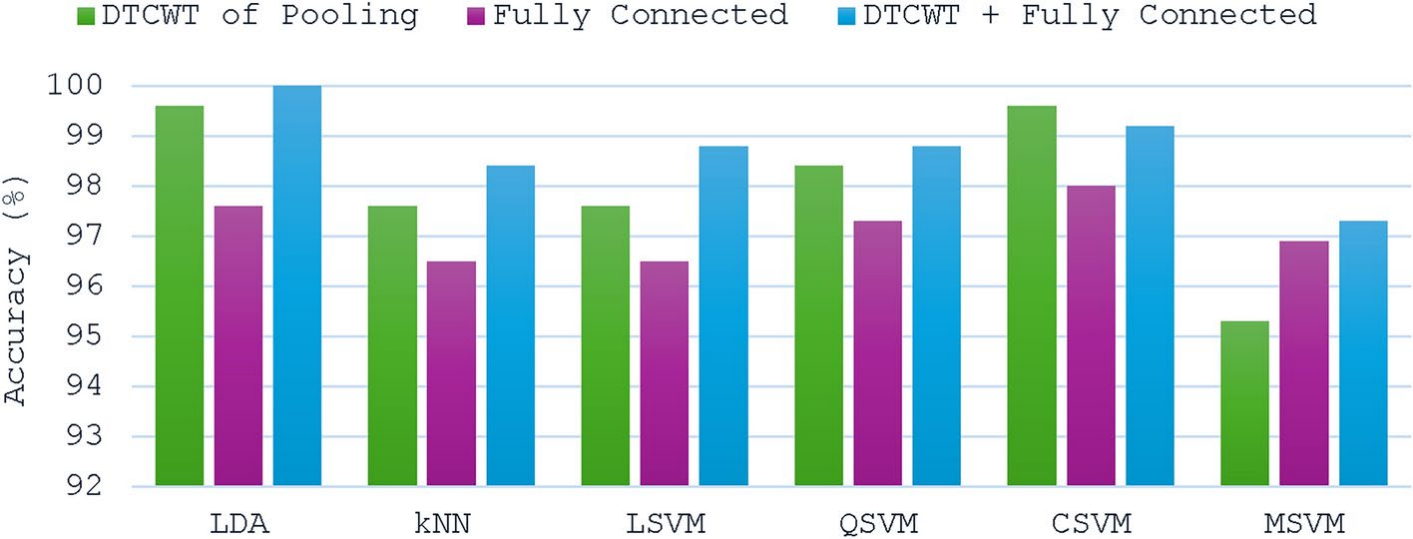
Accuracy results of the ResNet-18 CNN for the six considered classifiers when trained using features obtained from the pooling layer, fully connected layer, and combined DTCWT and fully connected layer.

This section presents the results of combining both the DTCWT features and the fully connected features of each CNN. These results are also compared with the DTCWT features and the fully connected features independently of each CNN and are shown in Figs. 3 and 4 for MobileNet and ResNet-18, respectively. Figure 3 shows that for the MobileNet CNN, the combined features of the DTCWT and fully connected layer attained higher accuracies (99.6%, 98.8%, 99.2%, 99.2%) than did the (99.2%, 97.6%, 98.4%, 98.8%) obtained by either the DTCWT features or fully connected features independently for the kNN, LSVM, CSVM and MSVM classifiers, respectively. However, for the LDA classifier, the combined features attained better accuracy (99.6%) than did the DTCWT features (98.8%), but the accuracy was the same as that of the fully connected features (99.6%). Finally, for the QSVM, the same accuracy of 98.8% is attained in the three feature scenarios. In short, the results shown in Fig. 3 prove that almost all classifiers merging features from two layers of MobileNet enhance performance compared to using features from a single layer.

For the ResNet-18 CNN, as shown in Fig. 4, the combined features of the DTCWT and the fully connected layer enhanced the performance of almost all the classifiers, especially for LDA, for which the performance reached 100%, whereas 99.6% of the features were attained solely by the DTCWT features and 97.6% of the fully connected layer features. Similarly, the combined features performed better than the fully connected layer features for the kNN (98.4%, 96.5%), LSVM (98.8%, 96.5%), QSVM (98.8%, 97.3%), CSVM (99.2%, 98.0%), and MSVM (97.3%, 96.9%). Similarly, the combined features had greater accuracy than did the DTCWT features for the kNN (98.4%, 97.6%), LSVM (98.8%, 97.6%), QSVM (98.8%, 98.4%), and MSVM (97.3%, 95.3%) classifiers. However, for the CSVM classifier, the combined features had slightly lower accuracy than the DTCWT features (99.2%, 99.6%). Generally, the accuracies demonstrated in Fig. 4 verify that integrating features from dual deep layers of ResNet-18 is superior to employing deep features from just one deep layer.

### Context III results

To verify the effectiveness of the feature selection stage, χ^2^ and ReliefF feature selection methods are employed separately to the combined deep features and selected features are employed to train considered classifiers. Tables 7 and 8 show accuracies attained using the six ML classifiers trained with various feature subsets selected by χ^2^ and ReliefF feature selection approaches using the combined deep features for MobileNet and ResNet-18, respectively.

**Table 7:** The accuracy (%) results for the MobileNet six classifiers trained with features selected by the χ^2^ method.

Number of features	LDA	kNN	LSVM	QSVM	CSVM	MSVM
Accuracy (%) using χ^2^ approach						
50	99.6	99.6	98.4	98.8	98.8	99.2
100	99.6	99.2	98.4	98.8	98.2	99.2
150	99.2	99.2	98.8	98.8	98.8	98.8
200	99.2	99.2	98.8	98.8	98.8	99.2
250	99.6	99.2	98.8	98.8	98.8	99.2
300	99.6	99.2	98.8	98.8	98.8	98.8
350	99.6	99.2	98.8	98.8	98.8	99.2
400	99.6	99.2	98.8	98.8	98.8	99.2
450	99.6	99.6	98.8	98.8	98.8	99.2
500	99.6	99.2	98.8	98.8	98.8	99.2
FULL (649)	99.6	99.6	98.8	98.8	99.2	99.2
Accuracy (%) using reliefF approach						
50	99.2	98.4	98.4	98.4	98.8	99.2
100	99.6	99.2	98.8	98.8	98.8	100
150	99.6	99.6	98.4	98.8	98.8	100
200	99.2	99.2	98.4	98.4	98.4	99.6
250	99.6	99.2	98.8	98.8	98.8	99.2
300	99.6	98.8	98.8	98.8	98.8	99.2
350	99.6	99.2	98.4	98.8	98.8	99.2
400	99.6	99.2	98.8	98.8	98.8	99.2
450	99.6	99.2	98.8	98.8	98.8	99.2
500	99.6	99.2	98.8	98.8	98.8	99.2
FULL (649)	99.6	99.6	98.8	98.8	99.2	99.2

**Table 8:** Accuracy (%) achieved for ResNet-18 six classifiers trained with features selected by χ^2^ and reliefF methods.

Number of features	LDA	kNN	LSVM	QSVM	CSVM	MSVM
Accuracy (%) using χ^2^ approach						
50	100	96.5	97.6	98.4	99.6	97.6
100	100	96.9	98.4	98.0	99.2	97.6
150	100	96.9	98.8	98.4	98.8	98.0
200	100	97.3	98.8	98.4	98.8	97.3
250	100	98.8	98.4	98.8	99.2	97.3
FULL (265)	100	98.4	98.8	98.8	99.2	97.3
Accuracy (%) using ReliefF approach						
50	100	96.5	98.0	98.4	98.8	97.3
100	100	98.0	98.8	98.8	99.2	98.0
150	100	98.8	98.4	98.8	99.2	98.0
200	100	98.8	98.4	98.8	99.2	98.0
250	100	98.8	98.4	98.8	99.2	98.0
FULL (265)	100	98.4	98.8	98.8	99.2	97.3

Table 7 demonstrates that using χ^2^ feature selection method, LDA (99.6%), kNN (99.6%), QSVM (98.8%), and MSVM classifiers (99.2%) achieved their peak accuracies using only 50 features similar to that attained by the full set of combined features of the MobileNet CNN (649 features). However, for the LSVM classifier, 150 features achieved the same accuracy (98.8%) as did the full set of MobileNet features. For the CSVM classifier, the highest accuracy (99.2%) was achieved using the full feature set only. Significantly, enlarging the feature count beyond 50 did not produce considerable enhancements, for LDA, kNN, QSVM, and MSVM, which achieved robust performance with accuracies between 98.8% and 99.6% using only 50 features, thus demonstrating that the χ^2^ method proficiently prioritized the most impactful features. Whereas, with MobileNet, the ReliefF feature selection strategy also showed good performance, although with somewhat less accuracy consistently across LDA, kNN, and QSVM classifiers for 50 features. With 100 and 150 features, respectively, LDA and kNN produced the highest accuracy of 99.6%, while QSVM attained 98.8% using 100 features. Nevertheless, LSVM attained 98.8% using 100 features, applying ReliefF, rather than the 98.8% attained using 150 features in the case of χ^2^ method. Finally, using the ReliefF approach, again CSVM achieved its highest accuracy of 99.2% using the full features set while the MSVM classifier achieved its 99.2% using 50 features as in the case of χ^2^ method. Small differences between ReliefF and χ^2^ methods’ results imply that the former prioritizes the most influential features, yet is not as efficient as χ^2^ approach. The latter is capable of reducing feature size used to train the ML models, and still maintains high accuracies which correspondingly lowers model complexity.

On the other hand, for the ResNet-18 CNN, Table 8 indicates that LDA could maintain 100% accuracy using only 50 features using either ReliefF or χ^2^ methods. Using 50 features, kNN and QSVM attained accuracies of 96.5% and 98.4% respectively again applying either ReliefF or χ^2^ methods. However, for 50 features size, CSVM, and MSVM achieved higher accuracies of 99.6% and 97.6%, respectively, using χ^2^ compared to the respective 98.8% and 97.3% achieved using the ReliefF method while only LSVM attained higher accuracy of 98% using ReliefF Approach compared to the 97.6% attained using χ^2^ method. Although the ReliefF method exhibited commendable performance overall, the χ^2^ method revealed a marginal advantage in enhancing accuracy across a number of classifiers with less feature count.

Conclusively, results, presented in Tables 7 and 8, show that χ^2^ method demonstrated superior performance over the ReliefF method regarding consistent accuracy throughout classifiers and feature subsets. Both MobileNet and ResNet-18 demonstrated that the χ^2^ method, in most cases, attained higher accuracy with fewer features, illustrating its greater ability to prioritize the most informative attributes while sustaining excellent fault diagnosis performance.

This effectiveness is especially beneficial for minimizing computational overhead in practical applications. Throughout all results, applying LDA with the ResNet-18 CNN, 100% accuracy is maintained using only 50 features.

Additional performance measures are calculated for the highest accuracy attained with the least number of features for each classifier highlighted in Tables 7 and 8. These metrics include precision, F1-score, specificity, and sensitivity and are shown in Tables 9 and 10 for MobileNet and ResNet-18, respectively.

**Table 9:** Performance measures of MobileNet classifiers trained with features selected by χ^2^ and reliefF approaches.

Classifier	Precision	Sensitivity	Specificity	F1-score
χ^2^ approach				
LDA	0.9961	0.9961	0.9995	0.9661
kNN	0.9961	0.9961	0.9995	0.9661
LSVM	0.9882	0.9882	0.9985	0.9882
QSVM	0.9882	0.9882	0.9985	0.9882
CSVM	0.9882	0.9882	0.9985	0.9882
MSVM	0.9921	0.9921	0.9990	0.9921
ReliefF approach				
LDA	0.9961	0.9961	0.9995	0.9661
kNN	0.9961	0.9961	0.9995	0.9661
LSVM	0.9921	0.9921	0.9990	0.9921
QSVM	0.9921	0.9921	0.9990	0.9921
CSVM	0.9921	0.9921	0.9990	0.9921
MSVM	0.9921	0.9921	0.9990	0.9921

**Table 10:** Performance measures of ResNet-18 classifiers trained with features selected by χ^2^ and reliefF methods.

Classifier	Precision	Sensitivity	Specificity	F1-score
χ^2^ approach				
LDA	1	1	1	1
kNN	0.9882	0.9882	0.9985	0.9882
LSVM	0.9882	0.9882	0.9985	0.9882
QSVM	0.9843	0.9843	0.9980	0.9843
CSVM	0.9961	0.9961	0.9995	0.9661
MSVM	0.9803	0.9803	0.9975	0.9803
ReliefF approach				
LDA	1	1	1	1
kNN	0.9882	0.9882	0.9985	0.9882
LSVM	0.9882	0.9882	0.9985	0.9882
QSVM	0.9882	0.9882	0.9985	0.9882
CSVM	0.9921	0.9921	0.9990	0.9921
MSVM	0.9803	0.9803	0.9975	0.9803

Table 9 shows that for the selected combined features of MobileNet, both LDA and kNN, applying either feature selection approach, have the same performance metric values, with a precision of 0.9961, sensitivity of 0.9961, specificity of 0.9995, and F score of 0.9661. Similarly, MSVM achieved a similar precision of 0.9921, sensitivity of 0.9921, specificity of 0.9990, and F score of 0.9921 with either selection approach. However, LSVM, QSVM, and CSVM classifiers had similar predictions of 0.9882, a sensitivity of 0.9882, a specificity of 0.9985, and an F score of 0.9882 using χ^2^ methodology rather than their precision of 0.9921, sensitivity of 0.9921, specificity of 0.9990, and F score of 0.9921 using ReliefF method. ReliefF method exhibited strong performance measures with MobileNet.

For the ResNet-18 selected features, Table 10 shows that LDA achieved the highest performance metric values of 1 for precision, sensitivity, specificity, and F1-score using either ReliefF or χ^2^ feature selection methods. Thus, this combination gives perfect performance measures and diagnosis accuracy with minimal feature size.

**Fig. 5:**
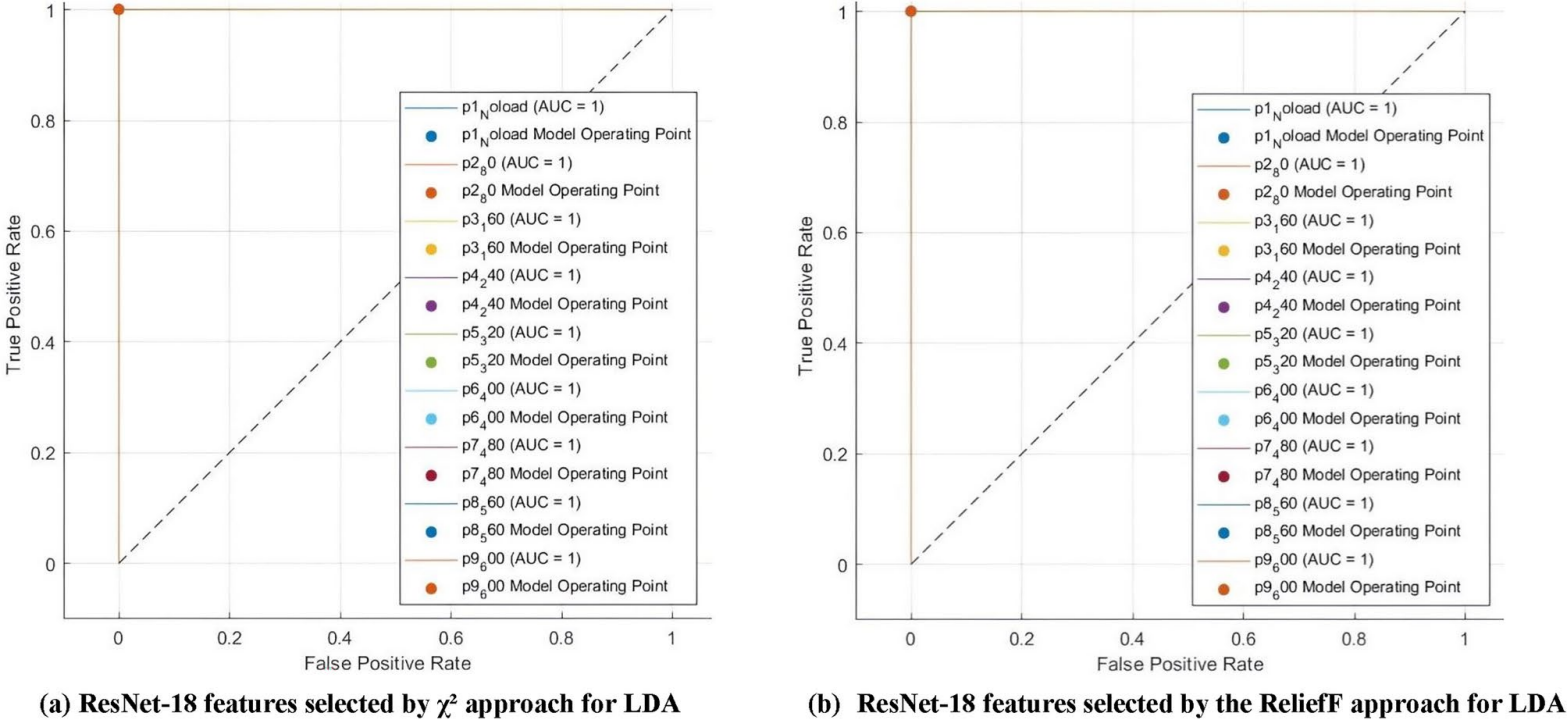
Confusion matrices of ResNet-18 features selected, to train LDA, using (**a**) χ^2^ approach, (**b**) Relief F approach.

**Fig. 6:**
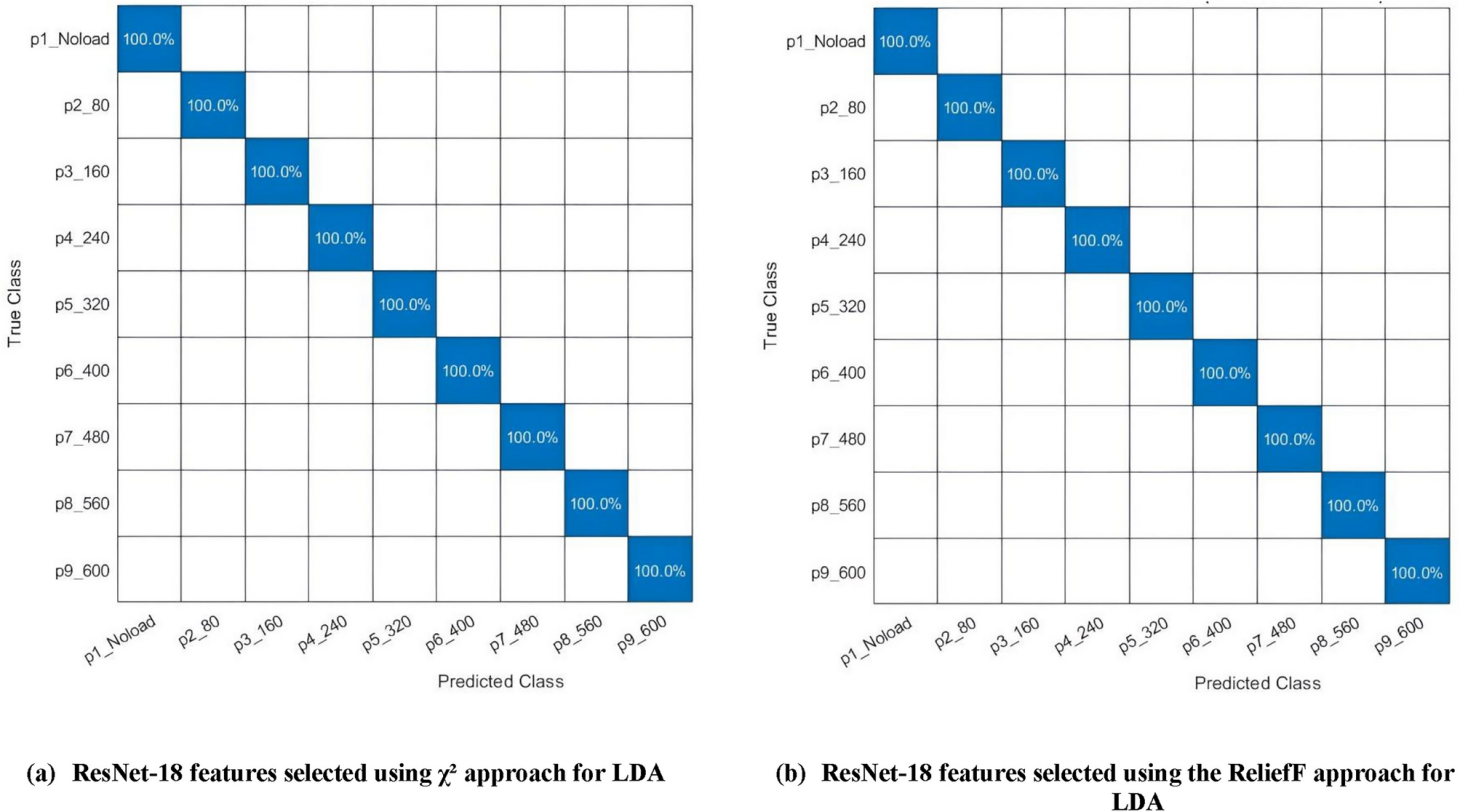
ROC curves and AUCs of ResNet-18 features selected, to train LDA, using (**a**) χ^2^ method, (**b**) Relief F method.

Conclusively, for ResNet-18 CNN, the LDA classifier demonstrated its resilience to various feature selection techniques by achieving perfect scores for precision, sensitivity, specificity, and F1-score at 100% along with its attained 100% diagnosis accuracy with a minimum number of features (50 features). This can be further confirmed using LDA confusion matrices shown in Fig. 5 which verify that the LDA classifier perfectly classified all the faults and classes in the dataset with a sensitivity of 100% using both feature selection methods. Moreover, in view of imbalanced classes, metrics such as receiving operating characteristics (ROC) and the area under the receiving operating characteristics curve (AUC-ROC) can provide more meaningful insights. Again, LDA ROC curves and AUCs, demonstrated in Fig. 6 show that it has an AUC of 1 for all 9 classes of the dataset using either selection technique.

**Fig. 7:**
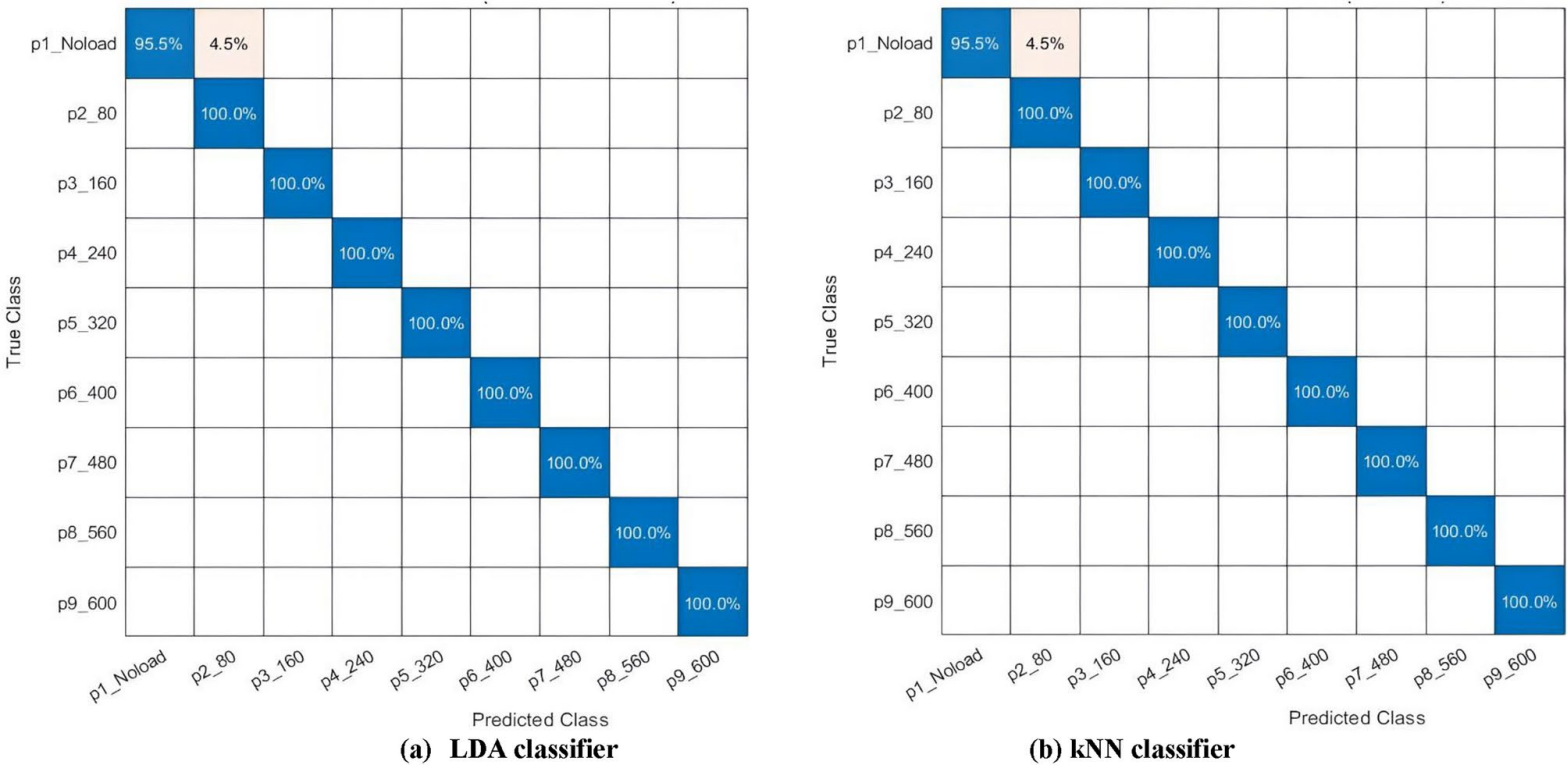
MobileNet features selected using χ^2^ approach to train (**a**) LDA classifier, (**b**) kNN classifier.

**Fig. 8:**
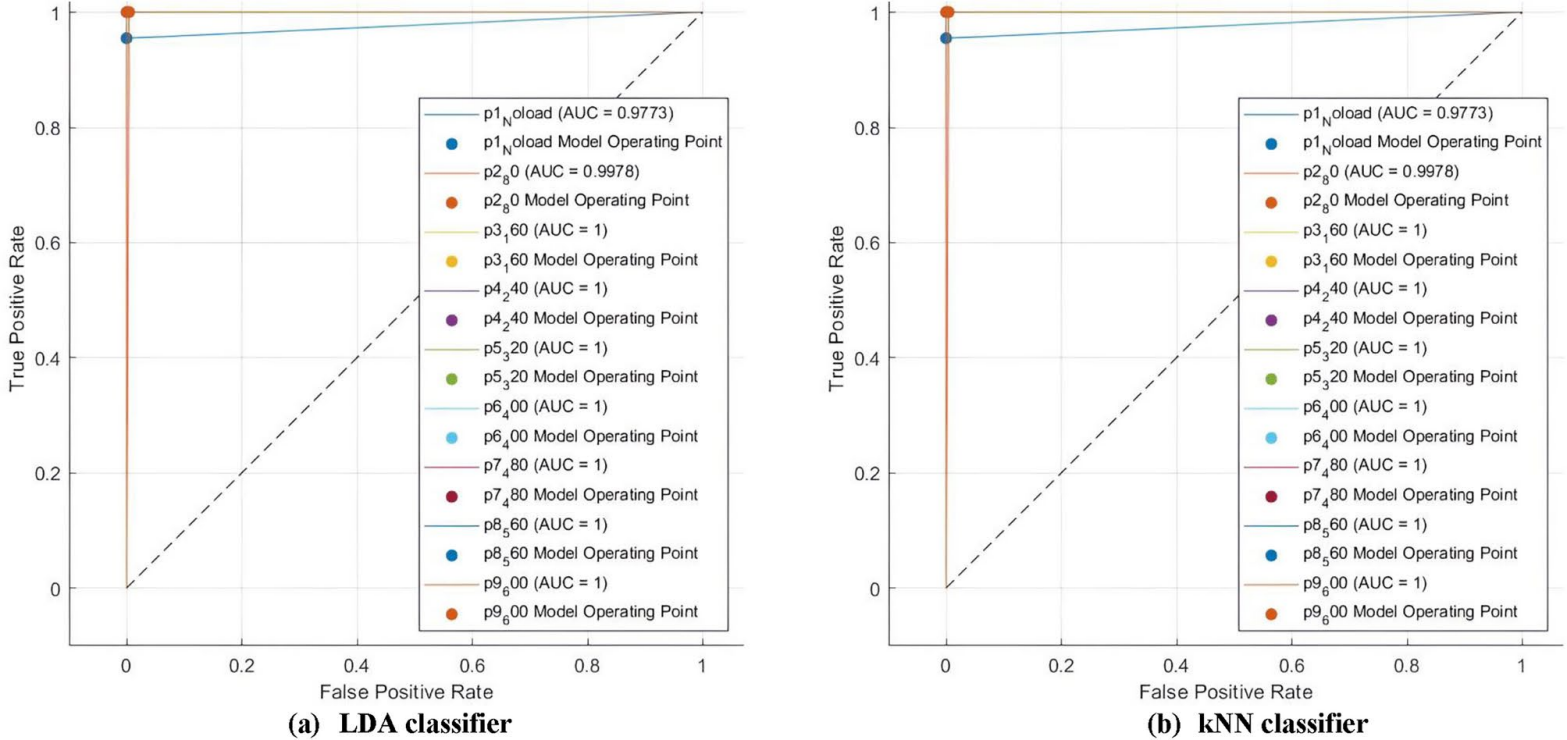
The ROC curves and AUCs of MobileNet deep features selected using χ^2^ approach to train (**a**) LDA, (**b**) kNN.

On the other hand, for MobileNet, the LDA and kNN classifiers attained peak accuracies of 99.6% using only 50 features when applying χ^2^ feature selection method rather than with the ReliefF approach. Therefore, their confusion matrices are presented in Fig. 7 where, for the MobileNet features selected by χ^2^ approach, both the LDA and kNN classifiers perfectly diagnosed all the faults with a sensitivity of 100%, yet the class with no load had a sensitivity of 95.5%. Regarding their ROC curves as well as the AUCs, Fig. 8 shows that both have an AUC of 1 for all faults but an AUC of 0.9773 for the no-load class.

Conclusively, Context III findings indicate the following:

- From Tables 7 and 8, χ^2^ method demonstrated superior performance over the ReliefF method regarding consistent accuracy throughout classifiers and feature subsets. Both MobileNet and ResNet-18 demonstrated that the χ^2^ method, in most cases, attained the highest accuracy with fewer features, illustrating its greater ability to prioritize the most informative attributes while sustaining excellent fault diagnosis performance. This effectiveness is especially beneficial for minimizing computational overhead in practical applications.
- Regarding peak accuracies across all results, ResNet-18 CNN with LDA classifier demonstrated robustness to all feature selection techniques by attaining 100% diagnosis accuracy and perfect scores for precision, sensitivity, specificity, and F1-score with a minimum number of features (50 features). On the other hand, for MobileNet, both LDA and kNN classifiers attained peak accuracy of 99.6%, using only 50 features when applying χ^2^ feature selection method rather than with the ReliefF approach.

## Discussion

This paper proposes a framework for detecting transformer inter-turn faults and diagnosing fault severity (i.e., the winding short-circuit ratio) using a DL-based thermography diagnostic method. The framework is based on two lightweight CNNs, MobileNet and ResNet-18, employing transfer learning, rather than relying on complex CNNs with large deep layers and parameters. Instead of extracting deep features from a single layer of a CNN, the suggested framework extracts features from dual deep layers of each CNN involving pooling and fully connected layers. The features of the pooling layers are still large in dimension; therefore, they are reduced using the DTCWT approach, which also represents time–frequency demonstrations of the input instead of depending only on spatial information. Finally, the suggested framework merges features obtained from the two layers of each CNN independently and then applies a feature selection approach to select the most important features and reduce the complexity of diagnosis.

Enhancements noted in the ablation study illustrate the cumulative effect of the suggested framework’s elements on overall performance. Every element—namely DTCWT, feature selection techniques, and integrated deep feature extraction—enhances both the performance of fault diagnosis and computational effectiveness. The findings of experimental work illustrate the contribution of each component in refining the framework to attain an optimal equilibrium between diagnosis accuracy and implementation complexity.

### Comparisons with different contexts of the suggested framework

The detection and diagnosis of the proposed framework are achieved through three steps. The initial context involves utilizing the deep features extracted from the fully connected layer of each CNN, along with the DTCWT variables derived from the pooling layer. These features are then separately input into six ML classifiers. In the subsequent context, the deep features from both deep layers are combined and employed as inputs for the same classifiers. In the third context, two feature selection techniques are used to choose attributes from the integrated dual deep layer features which are subsequently considered input into the six classifiers. Both χ^2^ and ReliefF feature selection methods gave very close performance results, yet with slightly better accuracies with χ^2^ method, hence the latter is selected.

**Fig. 9:**
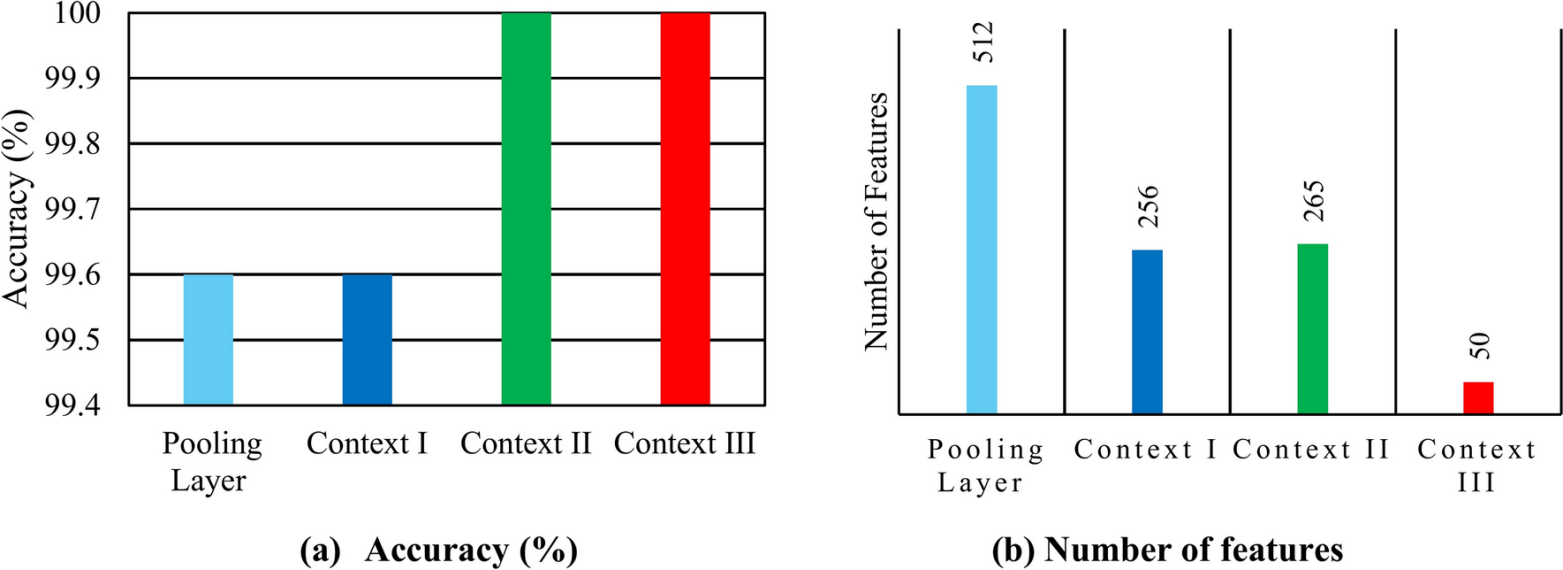
Peak accuracy and feature-size in each context for ResNet-18 applying LDA classifier and χ^2^ method.

Since ResNet-18 achieved the highest accuracy (100%), using the LDA classifier, a comparison between the highest accuracy and the number of features attained in each context for ResNet-18 is shown in Fig. 9. It can be noted that obtaining features from the pooling layer, using DTCWT in context I, maintained the 99.6% accuracy achieved by the pooling layer features yet at a smaller number of features (256 features using DTCWT compared to 512 features if it is not used) which verifies DTCWT effectiveness in balancing between diagnosis accuracy and complexity. Whereas context II has higher accuracy than of context I, thus verifying that merging deep features of DTCWT obtained from the pooling layer and the fully connected layer features has superior performance compared to using either pooling or fully connected features independently to train the ML model. Although context III has similar accuracy as that achieved in context II, this performance is attained with much fewer features (50 compared to 265).

The feature selection step led to better model interpretability which includes reducing model complexity, diagnosis time, and noise impact besides increasing model transparency and user expertise which is crucial for precise decision-making and on-time maintenance as follows:

- Mitigated Overfitting: A diminished set of pertinent features decreases the likelihood of overfitting, thereby ensuring the model generalises effectively to novel data. This is especially crucial in transformer fault diagnosis, where the objective is to precisely identify faults under diverse conditions.
- Improved model interpretability and transparency: Selecting the most relevant features will help set clearer cause-effect relationships that pinpoint which parameters are mainly affected by faults. Thus, the model’s behavior can be traced back to specific inputs, making it more transparent and understandable.
- Reduced model complexity: Selecting the most influential features focuses solely on variables that only matter most which reduces decision count and results in a more simplified decision-making process.
- Enhanced diagnosis speed: Removing less influential or redundant features reduces feature size which in turn reduces computation burden and fastens training and diagnosis processes.
- Reduced noise impact: Forcing the model to focus only on the most relevant data helps to exclude irrelevant or noisy features that can obscure the actual patterns related to faults.
- Domain expertise and fault prioritization: Selecting the most critical features for fault diagnosis allows experts to better decide which features should be monitored more closely and can prioritize maintenance actions based on the most important indicators.

The suggested framework’s feature selection method guarantees the retention of only the most significant features, resulting in a streamlined and effective model. This not only increases the precision of fault detection but also elevates the comprehension of the outcomes, thereby facilitating deeper insights into the fundamental fault mechanisms.

### Comparison with end-to-end DL models

A comparative analysis, between the diagnostic outcomes (accuracy and time) achieved by the end-to-end CNN models for the same dataset versus the findings obtained in context III of the suggested approach, is presented in Table 11 using χ^2^ approach for its superior performance. The analysis findings demonstrate that the suggested framework achieved a 100% accuracy rate using the LDA classifier trained with the selected features of ResNet-18, with the lowest training duration of 2.8509 s. Therefore, the proposed approach outperforms the most advanced CNN models, attaining accuracy of 100% (MobileNet, InceptionNet, Inception, ResNet-50, ResNet-101, Xception, and Shuffle CNN) since their training time is quite longer, ranging between 695 and 3982 s. The shortest computational time of 291 s was encountered by ResNet-18, yet with an accuracy of 89.90%.

**Table 11:** Results of end-to-end DL models compared to those attained in context III using χ^2^ approach.

Model	Accuracy (%)	Training time (sec)
Inception	100	1147
InceptionResNet	100	3982
MobileNet	100	644
ResNet-18	89.90	291
ResNet-50	100	790
ResNet-101	100	1335
Shuffle	100	695
Xception	100	753
Suggested framework models for MobileNet CNN χ^2^ approach		
LDA	99.6	7.3782
kNN	99.6	16.874
L-SVM	98.8	20.142
Q-SVM	98.8	13.451
C-SVM	98.8	13.24
M-SVM	99.2	12.947
Suggested framework models for ResNet-18 CNN using χ^2^ approach		
LDA	100	2.8509
kNN	98.8	3.0194
L-SVM	98.8	7.0037
Q-SVM	98.8	6.824
C-SVM	99.6	6.9524
M-SVM	98.0	9.51

Experimental results verify the ability of the proposed framework (with the ResNet-18 CNN and LDA classifiers) to attain optimal performance when combined with the proposed feature extraction and selection methods. This is concluded from the attained 100% accuracy and meanwhile its reduced computational complexity with the minimal number of features (50 features), thus minimizing the training time (2.85 s) and confirming its suitability for compact feature space compared to more sophisticated classifiers. This corresponds to the objective of attaining elevated classification accuracy while preserving computational efficiency, an essential factor for real-time, noninvasive fault detection in smart grid applications.

### Comparisons with related studies

The outcomes of the proposed system are compared to those of studies about the identification of winding faults in transformers using the same dataset of thermal images. The comparison is presented in Table 12, where the superiority of the suggested framework over other candidates is validated by the fact that it relies on a significantly smaller set of features that minimizes computational burden, training time, and classification complexity. Moreover, this approach avoids using a clustering or segmentation phase for diagnosis, thereby leading to further decreases in computational load and diagnostic time.

**Table 12:** Comparison with previous works involving the same thermogram image-based dataset.

References	Segmentation/clustering	# Classes	# Images	Features	Classifiers	Accuracy
^14^	Yes	9	255	3	AdaBoost and RF	95.6%
^17^	No	9	255	Large (2048)	SVM, ELM, DT, KNN, RF, ERT, SOF	100% for SVM
^18^	No	9	255	Large (2048)	Deep learning	100%
^19^	No	9	255	Large (2048)	Handcrafted CNN, VGG16, InceptionV3 and ResNet	100% for CNN
*Proposed	No	9	255	50	Light weight DL (ResNet-18)	100% for LDA

### Robustness of the proposed framework under different noises in IR images

Despite the importance of early real-time diagnosis of transformer faults to prevent catastrophic failures, traditional detection methods may face difficulties due to false alarms in case of non-fault fluctuation conditions. These conditions include noises in sensor data, transients during power changes, electromagnetic interference, and weather factors. However, these false alarms result in unnecessary maintenance and downtime, leading to significant operational inefficiencies and unneeded maintenance costs which should be avoided^56^. Thus, the robustness of the proposed framework, when dealing with noisy data, should be examined. To test the resilience of the proposed framework under different noise natures in the IR data, the same experiments were repeated, but under two types of noise, Gaussian and speckle applied to the IR data. Results indicated that although the framework sustains adequate performance, its diagnostic accuracy and classification efficacy were reduced compared to original noise-free conditions. This underlines the influence of noise on the framework’s performance:

- Outcomes of Context I: Under varying noise conditions, the framework’s performance exhibited a decline in diagnostic accuracy relative to noise-free data, with Gaussian noise inducing greater degradation than speckle noise owing to the former random intensity variations.
- Outcomes of Context II: Under Gaussian and speckle noise, the decline in accuracy was less significant than in Context I, showing superior noise management in this configuration due to merging more than one-layer features.
- Outcomes of Context III: Under both noises, despite a decline in accuracy within this context, the framework demonstrated considerable robustness in comparison to the other two contexts, especially with the ResNet-18 model.

#### Context I results under different noise

Table 13 clarifies the influence of noise on the fault diagnosis accuracy of the suggested framework in context I. Analysis of the outcomes under Gaussian and speckle noise conditions reveals that the framework’s efficiency varied markedly depending on the form of noise added as well as the classifier type and CNN model applied.

**Table 13:** The fault diagnosis accuracy (%) results using the six classifiers trained with DTCWT features and the fully connected features under different types of noise.

Features	Accuracy (%)					
	LDA	kNN	LSVM	QSVM	CSVM	MSVM
MobileNet						
Gaussian noise						
DTCWT	61.6	61.2	60.4	66.3	64.3	62.0
Fully connected	62.0	57.3	59.6	62.7	61.6	62.7
Speckle Noise						
DTCWT	67.8	69.0	69.8	72.2	70.2	66.3
Fully connected	67.1	62.7	69.4	64.7	66.3	68.2
ResNet-18						
Gaussian noise						
DTCWT	72.2	69.4	76.1	81.6	78.8	75.3
Fully connected	75.3	72.5	75.7	76.9	78.8	79.2
Speckle noise						
DTCWT	69.0	65.9	78.4	81.2	79.6	78.0
Fully connected	85.1	80.4	85.9	87.5	85.1	85.9

In the presence of Gaussian noise, classifiers attained accuracy levels ranging from 57.3% to 66.3% with MobileNet and from 69.4% to 81.6% with ResNet-18 where QSVM, with DTCWT extracted features**,** attained peak accuracy of 66.3% and 81.6% with MobileNet and ResNet-18 CNN models respectively. The decline in performance is due to random intensity fluctuations caused by Gaussian noise, which interfere with both spatial and frequency features. On the other hand, the framework exhibited better resilience to speckle noise, attaining accuracies among classifiers between 62.7% and 72.2% with MobileNet and between 65.9% and 87.5% with ResNet-18. Again, QSVM surpassed its competitors, attaining peak accuracies of 72.2% with MobileNet and DTCWT features and 87.5% with the ResNet-18 model and fully connected features.

These findings indicate that speckle noise, which predominantly impacts high-frequency components, exerts less effect on extracted features than Gaussian noise. Moreover, QSVM consistently exhibited superior robustness under both noise conditions. Finally, ResNet-18 outperformed MobileNet under noise conditions, presumably owing to its deeper architecture and capacity to discern more intricate patterns.

#### Context II results under different noise

**Fig. 10:**
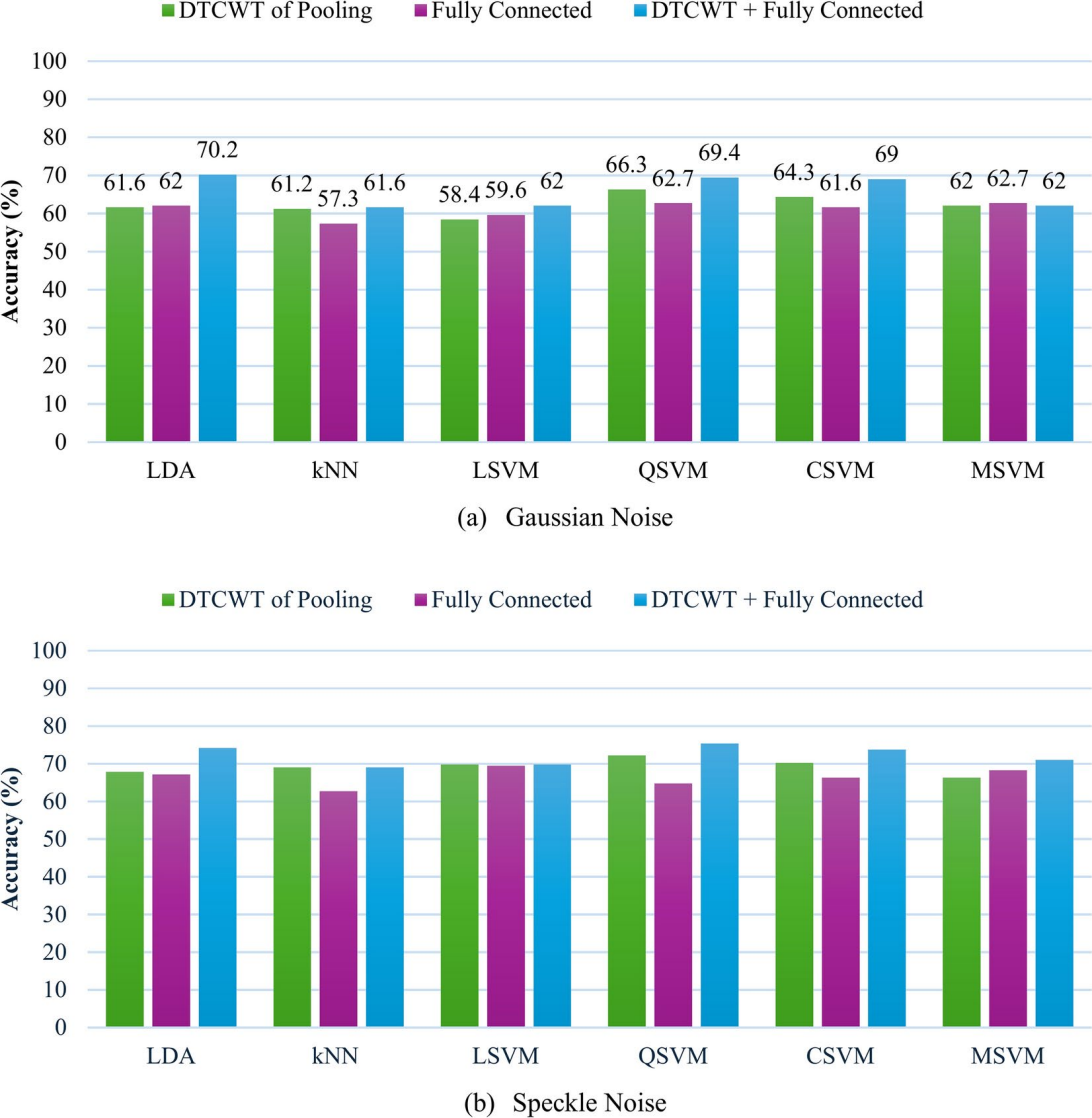
Accuracy of the MobileNet CNN for the considered six classifiers when trained using features obtained from DTCWT, fully connected layer, and combined features under different noise types: (**a**) Gaussian (**b**) Speckle.

**Fig. 11:**
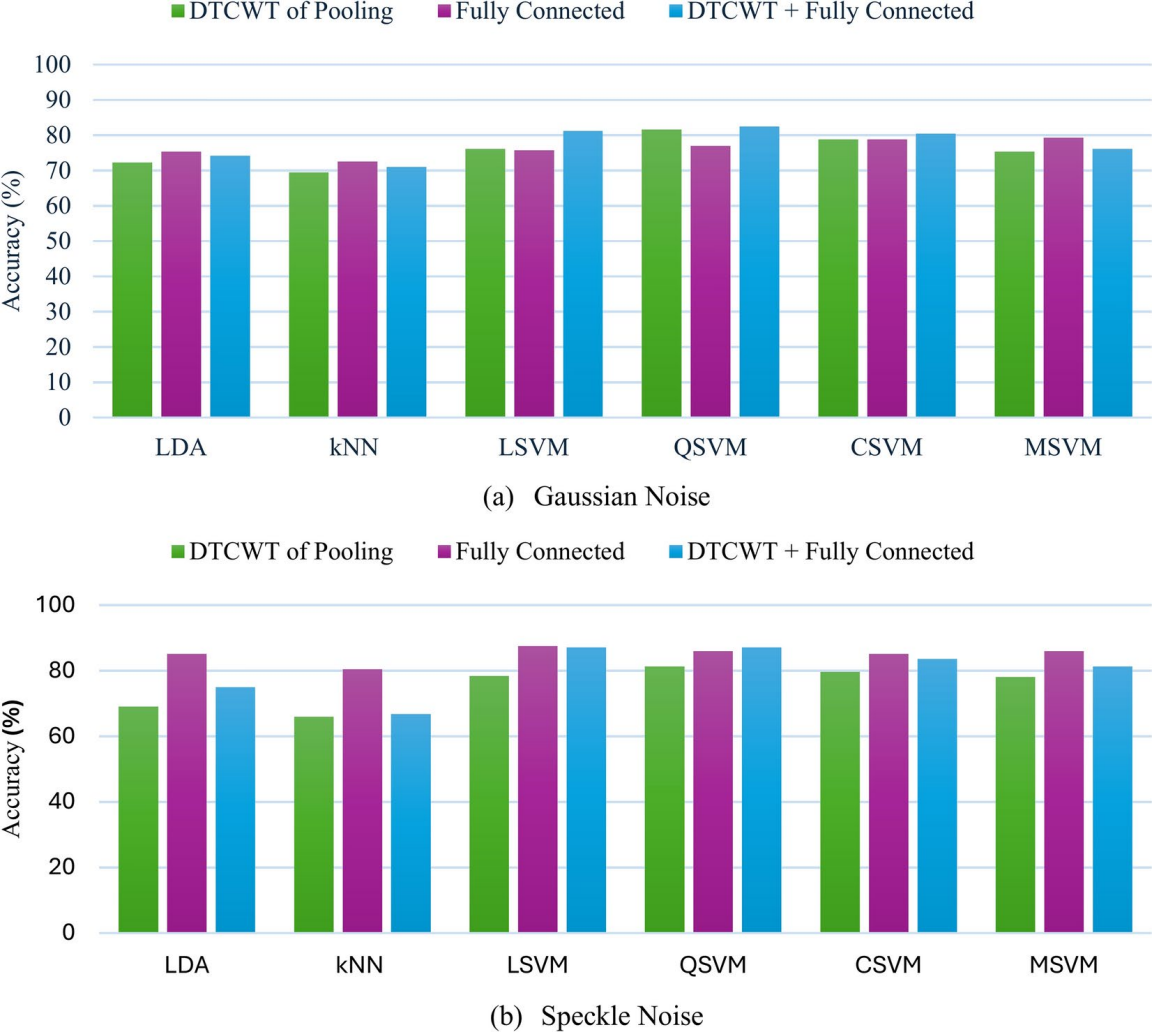
Accuracy of the ResNet-18 CNN for the six classifiers considered when trained using features obtained from DTCWT, fully connected layer, and combined features under different noise types: (**a**) Gaussian (**b**) Speckle.

Figures 10 and 11 for Context II offer a comprehensive assessment of the suggested framework’s efficacy under Gaussian and speckle noise, utilizing features from DTCWT of the pooling layer, fully connected layer, and combined DTCWT with a fully connected layer for classification. The study uncovers critical insights regarding the framework’s robustness and the efficacy of different CNN models, diverse classifiers along feature extraction procedures under various noise types. In the case of the MobileNet model undergoing Gaussian and speckle noise conditions, as shown in Fig. 10, merging DTCWT and FC features resulted in higher accuracies compared to those attained by single-layer features. Notably, among all classifiers, LDA and QSVM exerted the highest accuracies using the merged features enhancement, where LDA attained 70.2% and 74.1% under gaussian noise and speckle respectively whereas QSVM attained 69.4% and 75.3% under Gaussian and Speckle noises respectively. Thus, merging DTCWT with FC features enhanced diagnosis performance even under noise conditions.

On the other hand, for ResNet-18 as shown in Fig. 11, for some classifiers, integration of DTCWT and FC features leads to lower performance compared to that extracted from a single layer. Nevertheless, the highest accuracies were achieved using merged DTCWT and FC features in the case of QSVM which attained 82.4% under Gaussian noise and 87% under speckle noise.

Findings from Context II demonstrate the substantial benefits of combining DTCWT with FC features, with QSVM classifier, for both CNN models under different noise conditions where this classifier proved to be the most dependable, attaining the highest accuracy in both Gaussian and speckle noise conditions. Speckle noise typically exerted a lesser influence on classification accuracy than Gaussian noise. Finally, these results confirm the efficacy of the proposed framework in managing noisy conditions and offer insights into future improvements in fault diagnosis systems in real-world applications.

#### Context III results under different noise

The outcomes of Context III provide crucial information about how noise types, feature dimensionality, CNN model, and classifier choice affect the framework’s fault diagnosis accuracy. For the MobileNet model, as shown in Table 14, the QSVM classifier produced the highest accuracy of 74.1% with 500 features under Gaussian noise and an accuracy of 75.3% using all 649 features under speckle noise. In second place comes the LDA classifier with an accuracy of 72.2% and 74.5% with all 649 features under Gaussian and speckle noise respectively. On the other hand, ResNet-18 results, shown in Table 15, demonstrate that using a lesser number of features improves the fault diagnosis accuracy under both noise scenarios. With 50 features, QSVM had the highest accuracy for Gaussian noise (88.2%) and Speckle noise (89.4%), then came the LDA classifier with 50 features achieving 87.1% and 87.5% under each noise type respectively.

**Table 14:** Accuracy (%) results for the MobileNet six classifiers trained with different numbers of features selected by the χ^2^ feature selection method under different noise conditions.

Number of features	Accuracy (%)					
	LDA	kNN	LSVM	QSVM	CSVM	MSVM
Gaussian Noise						
50	65.5	65.5	67.5	70.2	67.8	65.5
100	61.2	66.7	68.6	71.0	71.4	68.6
150	56.5	65.5	66.3	69.0	70.6	65.5
200	45.9	64.7	64.3	68.6	71.4	66.7
250	60.0	65.5	65.5	70.6	71.2	65.5
300	62.4	65.9	64.3	70.6	70.2	65.9
350	63.5	65.5	65.1	72.2	71.0	63.9
400	67.1	64.3	63.5	73.3	70.6	65.1
450	65.9	63.9	62.7	73.3	71.4	64.7
500	65.9	62.7	64.3	74.1	71.8	63.5
649 (ALL)	72.2	61.6	62.0	69.4	69.0	62.0
Speckle Noise						
50	72.2	63.1	72.5	71.8	70.6	71.4
100	71.0	67.1	73.7	73.3	72.2	72.9
150	64.3	66.7	74.1	72.9	71.4	71.8
200	54.5	68.6	73.7	72.5	72.5	72.2
250	55.3	69.0	74.2	72.9	71.4	71.8
300	60.8	69.0	73.3	71.8	72.2	71.0
350	65.9	68.6	71.8	70.6	71.8	71.8
400	67.8	70.6	71.0	71.4	70.2	70.2
450	69.4	69.0	71.8	71.8	70.2	71.0
500	69.8	70.2	71.4	71	70.6	71.0
649 (ALL)	74.5	69.0	69.8	75.3	73.7	71.0

**Table 15:** Accuracy (%) results for the ResNet-18 CNN six classifiers trained with different numbers of features selected by the χ^2^ feature selection method under different noise conditions.

Number of features	Accuracy (%)					
	LDA	kNN	LSVM	QSVM	CSVM	MSVM
Gaussian Noise						
50	87.1	76.5	84.7	88.2	87.1	82.40
100	82.0	72.9	82.4	85.9	85.5	82.4
150	80.8	73.3	82.4	84.7	82.4	77.3
200	77.3	71.4	82.4	82.7	80.4	76.9
250	76.9	70.6	81.2	83.5	79.6	74.1
265 (ALL)	74.1	71.0	81.2	82.4	80.4	76.1
Speckle Noise						
50	87.5	76.9	89.4	89.4	87.1	85.9
100	82.4	72.5	89.4	87.5	85.9	85.9
150	78.4	70.6	87.8	87.5	86.3	83.9
200	77.6	71.0	87.5	87.5	85.1	83.1
250	74.5	67.1	86.7	86.7	84.7	81.6
265 (ALL)	74.9	66.7	87.1	87.1	83.5	81.2

Conclusively, Context III findings, under noise conditions, indicate that the following;

- Speckle noise exerted a lesser influence on classification accuracy than Gaussian noise, highlighting the necessity for noise-resistant feature extraction methods in real-world applications in the case of Gaussian noise owing to its random intensity variations and wider distribution and capacity to distort both high- and low-frequency image details.
- ResNet-18 outperformed MobileNet under noise conditions, presumably owing to the former’s deeper architecture and capacity to discern more intricate patterns.
- The impact of noise can be lessened by using a smaller, highly optimized feature subset, especially for classifiers that use decreased feature space’s discriminatory power, such as QSVM which continuously outscores other classifiers in both noise scenarios for both CNN models, yet with higher accuracy when being applied with ResNet-18.
- LDA classifier which gave superior diagnostic performance under noise-free conditions, was second in performance under different noise conditions with better accuracy again using the ResNet-18 model and χ^2^ feature selection approach with minimal feature size (only 50 features). In short, this proposed combination gives the best compromise in diagnostic performance under noise-free and noise-existing conditions.

### Complexity analysis of the proposed framework

The complexity of the suggested framework was evaluated by considering the two deployed CNN structures, ResNet-18 and MobileNet, as well as the classification phase as shown in Table 16.

**Table 16:** Complexity analysis of the suggested framework.

Methodology	Data aspects fed to classification algorithms	Deep network parameters amount	Deep layer count	Classification complexity (O)
ResNet-18	224 × 224 × 3	11 M	18	$O(k\bullet n\bullet d^2 )$^57^ *k:* kernel length *n:* The overall length of the pattern (amount of input entries) *d:* dimensionality of presentation
MobileNet	224 × 224x3	3.5M	28	
Fault diagnosis stage of the proposed framework	50 variables	LDA $(C-1)(p+1)$ *C:* category counts *p:* features length	–	$O(3/2 m\bullet p\bullet t+s/2 t^3$) ^58^ *p:* features length *m:* input instances count *s:* The mean quantity of non-zero attributes in a single example *t:* min (*m,p*)

ResNet-18 accepts inputs measuring 224 × 224 × 3 and contains approximately 11 million parameters distributed across 18 deep layers. The computational cost is denoted as *O(2kn*^*2*^*d)*, where *k* signifies the kernel length, *n* indicates the number of input entries, and *d* represents the dimensionality of the representation. MobileNet, conversely, is a more compact model comprising merely 3.5 million parameters across 28 layers, leading to diminished computational demands. During the fault diagnosis phase of the framework, classification is conducted on a diminished feature set of 50 attributes, thereby substantially reducing computational complexity. The computational complexity of the LDA classifier employed at this stage is denoted as *O(Cp),* where *C* represents the number of categories and *p* signifies the feature-length. Conclusively, low complexity emphasises the efficacy of the proposed method, especially in resource-limited settings, where the proposed framework leverages lightweight structures and optimized feature sets to achieve high classification accuracy while minimizing computational needs, rendering it ideal for real-time applications.

## Limitations of scalability and future directions

This work focuses on DL-based inter-turn fault diagnosis of small and medium-scale transformers applied in smart grids depending on the IR images dataset. However, with large-scale transformers, several challenges arise which need to be resolved to optimize the use of IRT for fault detection. These concerns remain the subject of several studies as the application of IRT for condition monitoring of electrical equipment develops and gains acceptance. Some of the future challenges to be addressed can be summarized as follows:

### Transformer construction challenges

Large power transformers are typically classified as oil-type and dry-type transformers. Despite both having the same principle of operation, the main difference lies in the inherent differences in construction, particularly their insulation, which imposes limitations on IRT application for fault detection. Oil-type transformers use synthetic oil as the primary insulating medium, whereas in dry-type ones, the windings are coated with solid insulating materials such as epoxy resin or cast resin. While IR imaging primarily detects surface temperature, this limitation is more experienced with oil-type transformers, where the oil can hide internal winding defects. For this reason, the fusion of multi-modal information in oil-type transformers is preferable over-relying on solely infrared images. Combining infrared thermography with other data modalities, such as dissolved gas analysis (DGA), not only improves fault classification but also helps in the precise identification of fault locations^59^. This can be done by converting the one-dimensional DGA data into three-dimensional feature images, thus enhancing the reliability of the condition monitoring system and facilitating image classification. However, despite the added benefit of high fault classification accuracy, it presents significant challenges related to data compatibility and model complexity, emphasizing the need for model compression and acceleration. Regarding dry-type transformer configuration, a notable problem arises in these transformers since they do not necessarily exhibit high temperatures during inter-turn faults, but they display distinct heat distribution patterns, especially when faults occur in different transformer phases^60^. Multiple fault traces for each phase are applied in^61^ followed by fault intensity adjustment. The process is repeated with all three phases of the transformer where data is sequentially organized according to variations in thickness and subsequently normalized. However, these modifications still come at the cost of increased complexity to the fault detection model.

### Feature extraction challenges

Since IR images are solely generated based on the heat distribution of an object, extracting the hot regions within an infrared image is challenging, especially when the image contains a very complex background and many interconnected systems. Thus, effective image segmentation is essential for isolating regions of interest in thermal images. For example, isolating transformers from the surrounding background could be made by estimating the average temperature of image pixels corresponding to the transformer and the protective fence using different segmentation approaches, such as simple linear iterative clustering and maximally stable extremal regions algorithm^62^. However, it is sometimes challenging due to some limitations with simple segmentation methods, which may result in over-segmentation or under-segmentation^63^. In^59^, the pyramid vision transformer (PVT) replaces the traditional approach of using different convolution strides to generate multi-scale feature maps. Again, the improvements in model accuracy come at the cost of increased model complexity, higher sensitivity to hyperparameter tuning, and longer training time^64^.

### Complex setups

In the context of electrical equipment detection, it is often hard to capture images with thermal cameras due to limited space availability^61^. For this reason, different sensors are placed at various angles to accommodate spatial constraints. Data fusion techniques and advanced architecture are thus required to be integrated in the condition monitoring framework, especially with large-scale three-phase transformers. However, other challenges may arise such as large processing time due to the fusion of large volumes of data and possible redundancy and overlapping of images which may complicate the detection process^65^.

### Noise and image quality

Large-scale transformers are often located outdoors, making the captured image more affected by noises, non-uniformity, and environmental factors such as wind, humidity, and ambient temperatures. As demonstrated in section "Robustness of the proposed framework under different noises in IR images", the presence of these factors not only complicates the analysis and interpretation of thermal data but also affects the accuracy of fault detection^66^. To overcome this problem, images are pretreated with image quality enhancement techniques^63^. For contrast enhancements and environmental effects reduction, adopting various filtering techniques can filter noisy data, such as basic filtering approaches (mean, median, and adaptive filtering), advanced filtering algorithms such as wavelet transform^67^, and histogram equalization techniques^68^.

On the other hand, noise-tolerant DL models can ensure accurate and reliable fault diagnosis even in the case of noisy, incomplete, or corrupted data^69^. Since these models are less sensitive to noise, they can respond fast and effectively in dynamic environments, offering real-time diagnosis without requiring additional data cleaning or preprocessing. When a model is trained with noise-tolerant techniques, it is able to learn patterns in the data, focusing on the most influential features for fault detection, despite the presence of noises. This enhances the model’s fault diagnosis accuracy and robustness as well as improves its generalization capabilities to handle noisy data. Noise-tolerant models can be achieved by applying denoising techniques to convolutional neural networks (DCNN)^70^. This improves extraction clarity, leading to a more robust process for fault detection and discrimination, yet at the cost of significant need of computational requirements due to the more added layers to the CNN model^71^. Alternatively training noise-tolerant models, with synthetic noisy data or noise augmentation techniques, as well as adopting adaptive learning algorithms can help DL models distinguish between noises or normal variances and actual faults more effectively^72^.

Moreover, employing sophisticated preprocessing methods, such as wavelet denoising, could substantially mitigate the influence of noise on the input pictures prior to their introduction into the CNN. Within our system, the dual-DTCWT not only diminishes the size of deep features but also intrinsically offers a degree of noise attenuation by encapsulating both spatial and time–frequency information. This dual representation enables the model to concentrate on specific patterns while eliminating extraneous noise elements. Ultimately, ensemble learning methods, which involve training multiple models and aggregating their predictions, can significantly improve the system’s robustness. Ensemble approaches may efficiently diminish the impact of noise and enhance overall classification accuracy by integrating outputs from various models.

## Conclusion

Ensuring power transformers’ reliability in smart grids minimizes economic disruptions and contributes to the attainment of sustainable development goals by promoting affordable and clean energy and supporting sustainable industrialization. In this paper an online thermogram image-based DL method is proposed for early detection of transformer winding faults as well as diagnosis of fault severity with minimal interruption to transformer operation, thus maximizing energy saving and system economics. Unlike the huge feature number exhibited by previous related studies involving the same dataset, the proposed approach uses minimal features, thus reducing classification complexity and time while maintaining 100% classification accuracy. This is achieved owing to the modifications proposed in the applied approach which include; (i) adopting DTCWT to reduce the huge size of extracted features as well as obtain a time–frequency demonstration besides the spatial deep features, (ii) Features extracted from CNN pooling layer using DTCWT are merged with featured extracted from CNN fully connected layers to integrate both layers’ knowledge thus enhancing diagnosis accuracy, (iii) a feature selection process is applied to the combined features to reduce features’ dimension, thus minimizing classification complexity and training time while maintaining classification accuracy. Finally, no clustering or segmentation phases are required in the proposed technique, resulting in a further decrease in implementation complexity. Under noise-free conditions, experimental results verify that the proposed framework relying on ResNet-18 CNN could achieve 100% accuracy with only 50 features selected using χ^2^ feature selection approach to train the LDA classifier for a minimal training time of 2.8509 s. Moreover, the proposed diagnosis approach robustness is validated under Gaussian and Speckle noises, where results confirmed that again the proposed combination of ResNet-18 CNN model, LDA classifier, and the χ^2^ method comes second place in enhancing class accuracy in low-dimensional spaces (50 features) i.e. achieving accuracies of 87.1% and 87.5% under each noise type respectively. Thus, this combination achieves the best compromise in diagnosis performance under different noise-free and noise-existing conditions. Yet, to achieve higher accuracies during noise conditions, it is recommended to add filtering techniques and noise-resistant feature extraction methods in future work, especially for large-scale transformers kept outdoors.

## Data Availability

The data employed in this study can be found at the following link**:** https://data.mendeley.com/datasets/8mg8mkc7k5/3 (accessed 10 January 2024)
